# Targeting the SIN3A-PF1 interaction inhibits epithelial to mesenchymal transition and maintenance of a stem cell phenotype in triple negative breast cancer

**DOI:** 10.18632/oncotarget.6048

**Published:** 2015-10-09

**Authors:** Nidhi Bansal, Kevin Petrie, Rossitza Christova, Chi-Yeh Chung, Boris A. Leibovitch, Louise Howell, Veronica Gil, Yordan Sbirkov, EunJee Lee, Joanna Wexler, Edgardo V. Ariztia, Rajal Sharma, Jun Zhu, Emily Bernstein, Ming-Ming Zhou, Arthur Zelent, Eduardo Farias, Samuel Waxman

**Affiliations:** ^1^ Division of Hematology and Oncology, The Tisch Cancer Institute, Icahn School of Medicine at Mount Sinai, New York, USA; ^2^ Division of Clinical Studies, Institute of Cancer Research, Sutton, United Kingdom; ^3^ Department of Oncological Sciences, Department of Genetics and Genomic Sciences, Graduate School of Biomedical Sciences, Icahn School of Medicine at Mount Sinai, New York, USA; ^4^ Genetics and Genomic Science, Icahn School of Medicine at Mount Sinai, New York, USA; ^5^ Department of Oncological Sciences, Graduate School of Biomedical Sciences, Icahn School of Medicine at Mount Sinai, New York, USA; ^6^ Division of Hemato-Oncology, Department of Medicine, Sylvester Comprehensive Cancer Center, Miller School of Medicine, University of Miami, Florida, USA

**Keywords:** epigenetics, SIN3, PF1, triple negative breast cancer, cancer stem cells

## Abstract

Triple negative breast cancer (TNBC) is characterized by a poorly differentiated phenotype and limited treatment options. Aberrant epigenetics in this subtype represent a potential therapeutic opportunity, but a better understanding of the mechanisms contributing to the TNBC pathogenesis is required. The SIN3 molecular scaffold performs a critical role in multiple cellular processes, including epigenetic regulation, and has been identified as a potential therapeutic target. Using a competitive peptide corresponding to the SIN3 interaction domain of MAD (Tat-SID), we investigated the functional consequences of selectively blocking the paired amphipathic α-helix (PAH2) domain of SIN3. Here, we report the identification of the SID-containing adaptor PF1 as a factor required for maintenance of the TNBC stem cell phenotype and epithelial-to-mesenchymal transition (EMT). Tat-SID peptide blocked the interaction between SIN3A and PF1, leading to epigenetic modulation and transcriptional downregulation of TNBC stem cell and EMT markers. Importantly, Tat-SID treatment also led to a reduction in primary tumor growth and disseminated metastatic disease *in vivo*. In support of these findings, knockdown of *PF1* expression phenocopied treatment with Tat-SID both *in vitro* and *in vivo*. These results demonstrate a critical role for a complex containing SIN3A and PF1 in TNBC and provide a rational for its therapeutic targeting.

## INTRODUCTION

Breast cancer is a complex and heterogeneous disease with diverse molecular and clinical phenotypes. The molecular subtyping of breast cancer is broadly based on the status of estrogen receptor (ER), progesterone receptor (PR) and epidermal growth factor receptor 2 (Her2) [1]. Triple negative breast cancer (TNBC), an aggressive subtype comprising 15-20% of breast cancer incidences is associated with early recurrence, shorter median survival time after relapse and development of chemoresistant disease. [2]. Despite considerable therapeutic advances for ER-positive and Her2-positive breast cancers, targeted drugs are not yet clinically available for TNBC [2] and their future development will require a better understanding of the biology of TNBC tumors.

Although 70% of TNBC tumors phenotypically resemble basal-like breast cancer and genetic mutations at the *BRCA1* and *TP53* loci are frequently observed [3], molecular profiling suggests that TNBC is in fact a heterogeneous entity. This heterogeneity in TNBC (and cancer in general), cannot be explained by classic genetics alone and it has become increasingly clear that aberrant epigenetics play a significant role [4]. Tumor-associated changes in methylation of DNA or core histones H3 and H4, resulting in deregulated expression of important TNBC genes including *ESR1*, *CDH1*, *MUC1* and *BRCA1,* have been attributed to development of TNBC [4]. Aberrant epigenetics also underpin the cellular plasticity required for the functional adaptation of cancer cells to their environment, including epithelial-to-mesenchymal transition (EMT) that is necessary for the tumor invasion-metastasis cascade and acquisition or maintenance of stem cell-like traits of tumor-initiating cancer stem cells (CSCs) [5]. The reversible nature of epigenetic changes, however, also presents opportunities for therapeutic intervention and numerous “epidrugs”, including histone deacetylase inhibitors and demethylating agents, are being evaluated in TNBC [6-9].

Epigenetic reconfiguration in cancer cells is brought about by aberrant recruitment of chromatin modifying complexes, including the SIN3 transcription complex, that possess diverse chromatin modifying enzymatic activities. Mammalian SIN3 (SIN3A and SIN3B), via its multiple protein-protein interaction domains, serves as a scaffold bridging together sequence-specific DNA binding transcription factors and various chromatin regulators [10, 11]. Both SIN3A and SIN3B are characterized by a unique arrangement of four paired amphipathic α-helix (PAH1-PAH4) motifs. While they share sequence homology, the different PAH domains mediate specific SIN3A and SIN3B interactions, with the second PAH repeat (PAH2) reported to bind a functionally diverse group of proteins, including the MAD family of repressors, that contain a motif known as a SIN3 interaction domain (SID) [12]. The SIN3 complex has important regulatory functions in cell proliferation, development and differentiation and its aberrant recruitment is implicated in breast cancer pathogenesis [13-15].

Our previous work has suggested that blocking specific interactions of the SIN3 PAH2 domain could represent a novel therapeutic approach in TNBC [15, 16]. Using a peptide corresponding to the SID domain of MAD (Tat-SID) we sought to characterize the phenotypic consequences of interfering with SIN3 function and identify candidate PAH2-interacting factors in TNBC. Here, we report the identification of the SID-containing adaptor protein PF1 (PHF12), which is expressed from a locus amplified in breast cancer [17, 18], as a factor required for EMT and cancer stem cell maintenance in TNBC.

## RESULTS

### Tat-SID disrupts the interaction between SIN3A and a complex containing PF1, MRG15 and KDM5B

**Figure 1:**
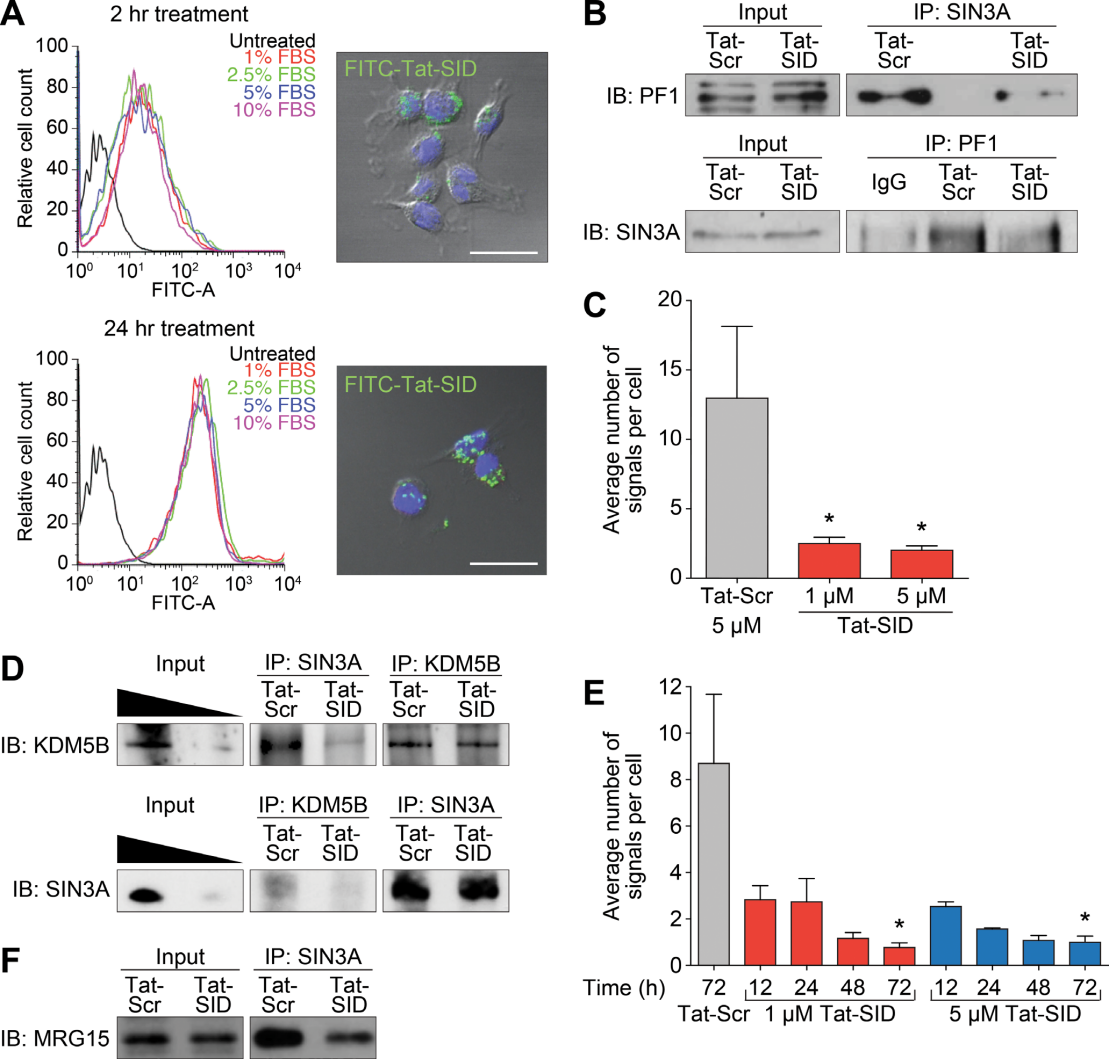
Tat-SID disrupts interaction between SIN3A and a chromatin regulating protein complex containing PF1, MRG15 and KDM5B **A.** Left, histograms showing cellular uptake of Tat-SID analyzed by flow cytometer in MDA-MB-231 cells treated with 1 μM FITC-conjugated Tat-SID for 2 h and 24 h in 1% (red), 2.5% (green), 5% (blue) and 10% (pink) serum. Black histogram represents untreated cells. Right, confocal images of MDA-MB-231 treated with 1 μM FITC-conjugated Tat-SID (green) and stained with nuclear stain DAPI (blue). Scale bars: 25 μm. **B.** IP-immunoblot analysis of MDA-MB-231 cells treated with 1 μM Tat-SID for 24 h, immunoprecipitated with anti-Sin3A antibody and immunoblotted with anti-PF1 antibody (upper panel), or immunoprecipitated with anti-PF1 antibody and immunoblotted with anti-Sin3A antibody (lower panel). Input corresponds to 10% of the total protein used for immunoprecipitation. **C.** Quantification of proximity ligation assay (PLA) analyzing the interaction between SIN3A and PF1 in MDA-MB-231 cells treated with 1 μM and 5 μM Tat-SID treatments respectively for 24 h (red) in comparison to Tat-Scr (grey). Tat-Scr versus 1 μM Tat-SID, *, *p* = 0.0251; Tat-Scr versus 5 μM Tat-SID, *, *p* = 0.0217; p, unpaired *t*-test. **D.** IP-immunoblot analysis of MDA-MB-231 cells treated with 1 μM Tat-SID for 24 h, immunoprecipitated with anti-Sin3A & anti-KDM5B antibodies and immunoblotted with anti-KDM5B antibody (upper panel), or immunoprecipitated with anti-KDM5B & anti-Sin3A antibodies and immunoblotted with anti-Sin3A antibody (lower panel). Input corresponds to 20% and 5% of the total protein used for immunoprecipitation. **E.** Quantification of PLA analyzing the interaction between SIN3A and KDM5B in MDA-MB-231 cells treated with 1 (red) and 5 μM Tat-SID (blue) for different time points in comparison to 5 μM Tat-Scr (grey). Tat-Scr versus 1 μM Tat-SID 72 h, *, *p* = 0.0119; Tat-Scr versus 5 μM Tat-SID 72 h, *, *p* = 0.0111; p, unpaired *t*-test. **F.** IP-immunoblot analysis of MDA-MB-231 cells treated with 1 μM Tat-SID for 24 h, immunoprecipitated with anti-Sin3A antibody and immunoblotted with anti-MRG15. Input corresponds to 10% of the total protein used for immunoprecipitation. Error bars represent mean ± SD (*n* = 3).

The PAH2 domain of SIN3 mediates interactions with a restricted subset of factors containing a conserved SIN3 interaction domain (SID) with homology to amino acids 5-24 of the prototypic PAH2-binding protein, the transcriptional repressor MAD [19-22]. In order to dissect the function of SIN3 PAH2 we used a 31-mer decoy peptide comprising amino acids 5-24 of MAD SID and the nuclear localization signal of HIV-1 Tat (Tat-SID: YGRKKRRQGGG-VRMNIQMLLEAADYLERRER), which results in increased nuclear accumulation of Tat-SID decoy peptide with time irrespective of serum concentration (Figure 1A). We focused our investigation on the plant homeodomain (PHD)-containing protein PF1 (PHF12), which links SIN3 PAH2 to a chromatin-modifying protein complex containing MRG15, LID (the *Drosophila* homolog of KDM5A/B) and EMSY [20, 23-25] that has been implicated in breast cancer [26-30]. Consistent with our previous results demonstrating Tat-SID-mediated disruption of the interaction between PAH2 and SID-containing MAD [15] (Figure S1), both co-immunoprecipitation and proximity ligation assay (PLA) showed that Tat-SID effectively blocked the SIN3A-PF1 interaction in MDA-MB-231 cells (Figures 1B, 1C and S2A). In comparison to MAD, PF1 has been reported to bind to PAH2 with a 10-fold lower affinity [31] and we also found the amount of a peptide corresponding to the PF1-SID required to compete for binding to a FITC-labeled MAD probe to be 12-fold greater (IC_50_ = 1.26 μM for MAD SID versus 15.59 μM for PF1 SID; Figure S2B). EMSY has also recently been identified as a binding partner for KDM5B [29], which prompted us to investigate the interaction between KDM5B and SIN3A. Supporting the notion that SID treatment could disrupt a functional complex, the interaction between SIN3A and KDM5B was also inhibited in a time- and concentration-dependent manner (Figures 1D, 1E and S2C). A decrease in the association between SIN3A and MRG15 following Tat-SID treatment was also observed, although this effect was less pronounced and may be due to the presence of an additional MRG15 binding site in the histone interaction domain (HID) of SIN3 (Figure 1F) [23].

### Blocking SIN3-PAH2 interactions inhibits the EMT program in cancer cells

**Figure 2:**
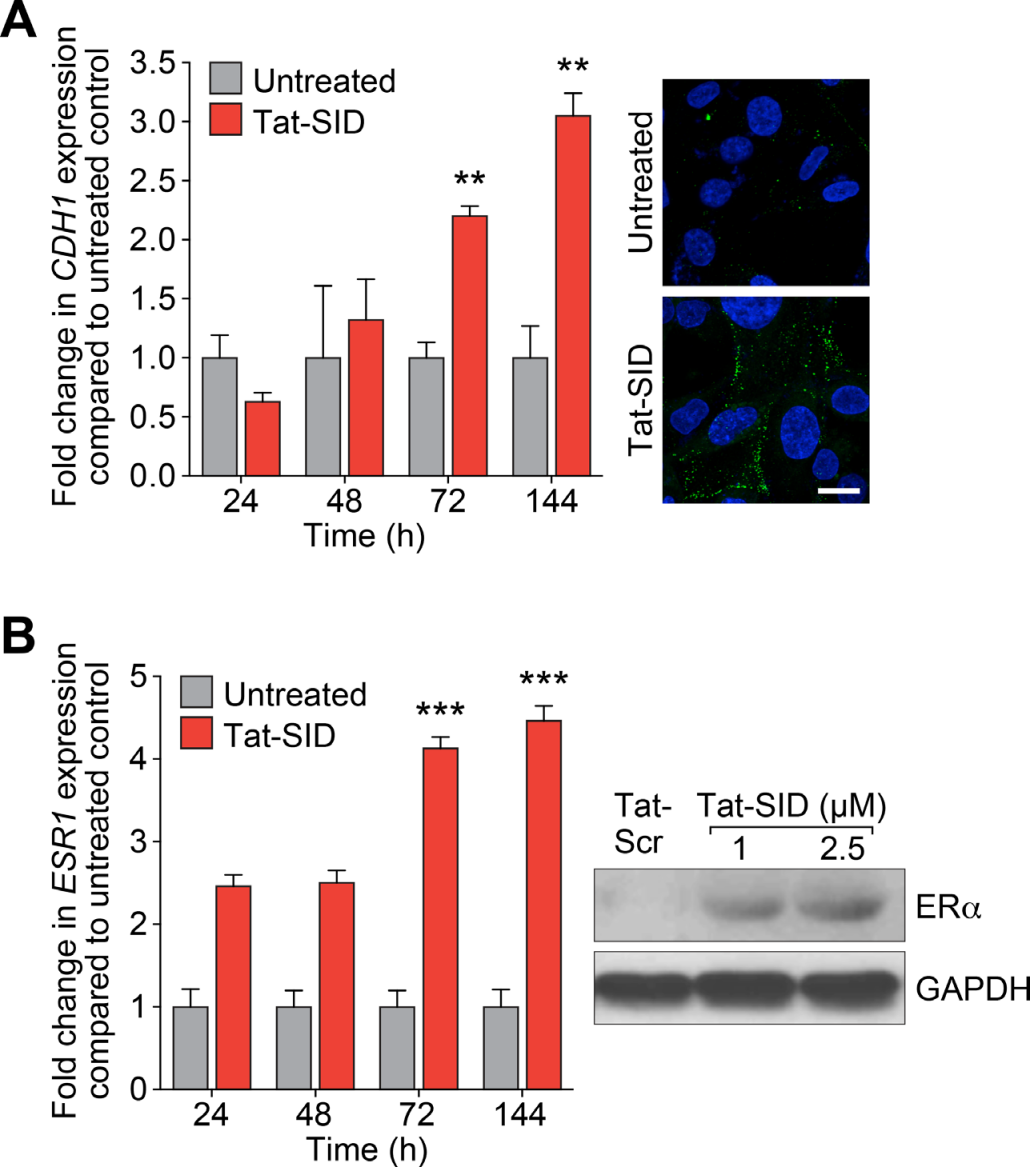
Tat-SID induces functional re-expression of epigenetically silenced *ESR1* and *CDH1* **A.** Left, qRT-PCR for expression of *CDH1* mRNA in MDA-MB-231 cell untreated (grey) or treated (red) with 2.5 μM Tat-SID for the indicated time. Untreated versus Tat-SID 72 h, **, *p* = 0.0016; untreated versus Tat-SID 144 h, **, *p* = 0.0035; p, unpaired *t*-test. Right, confocal images of MDA-MB-231 cell untreated or treated with 2.5 μM Tat-SID for 72 h and immunostained for CDH1 protein (green). Blue is the nuclear stain DAPI. Scale bar: 20 μm. **B.** Left, qRT-PCR for expression of *ESR1* mRNA in MDA-MB-231 cell untreated (grey) or treated (red) with 2.5 μM Tat-SID for the indicated time. Untreated versus Tat-SID 72 h, ***, *p* = 0.0002; untreated versus Tat-SID 144 h, ***, *p* = 0.0002; p, unpaired *t*-test. Right, immunoblot analysis of ER*α* expression in MDA-MB-231 cells treated with Tat-Scr or Tat-SID for 7 days.

Consistent with our previous results obtained by stably expressing SID peptide [15], treatment with Tat-SID led to a time-dependent increase in expression of *CDH1* mRNA of greater than 3-fold and plasma membrane-associated E-cadherin became evident at 72 h (Figure 2A). Similar results were observed for ERα with 4.5-fold increase in *ESR1* expression and increased protein levels after 7 days of Tat-SID treatment (Figure 2B). SID decoy was also found to induce re-expression of *CDH1* and *ESR1* in three additional TNBC lines, MDA-MB-157, 4T1 and MMTV-Myc (Figure S3A and S3B). To gain further insight into transcriptional reprogramming associated with Tat-SID treatment, we performed expression microarray analysis. Pathway analysis of these data identified regulation of EMT as one of the most significant pathways modulated in Tat-SID treated cells compared to Tat-Scr (Table 1 and Tables S1-S3). Other pathways that were significantly regulated included cell migration/cell adhesion, cell proliferation and cell death and survival (Tables S2 and S4). Of note, Tat-SID treatment induced downregulation of important molecular markers of EMT such as *FGFR2*, *FGFR4*, *TWIST1* and *WNT5A* (Table 1). Of these, Tat-SID induced down-regulation of *FGFR2, FGFR4* and *WNT5A* were validated by qRT-PCR (Figure S4). Further evidence of Tat-SID-induced regulation of EMT was provided by the ‘Upstream transcription factor analysis’ that predicted inhibition of *TGFB1* (z score: −4.4), *CTNNB1* (β-catenin) (z score: −3.3), *SMAD3* (z Score: −2.6) and *SMAD4* (z score: −2.2), four major inducers of EMT (Tables S5 and S6). Other genes encoding relevant transcription factors predicted to be downregulated upon Tat-SID treatment included *RARG*, *MAPK3* and *E2F1*, offering additional clues to the mechanisms underlying inhibition of cell proliferation and migration pathways (Table S2).

**Table 1:** List of genes involved in EMT that are downregulated in MDA-MB-231 cells treated with Tat-SID

Gene	Entrez Name	Log Ratio (Tat-SID/Tat-Scr)
*CDH2*	Cadherin 2, type 1, N-cadherin (neuronal)	−1.480
*EGR1*	Early growth response 1	−1.050
*FGF2*	Fibroblast growth factor 2 (basic)	−1.510
*FGFR2*	Fibroblast growth factor receptor 2	−2.040
*FGFR4*	Fibroblast growth factor receptor 4	−1.330
*FOXC2*	Forkhead box C2 (MFH-1, mesenchyme forkhead 1)	−1.370
*PIK3C2B*	Phosphatidylinositol-4-phosphate 3-kinase, catalytic subunit type 2 beta	−1.230
*TWIST1*	Twist basic helix-loop-helix transcription factor 1	−1.010
*WNT10B*	Wingless-type MMTV integration site family, member 10B	−1.050
*WNT5A*	Wingless-type MMTV integration site family, member 5A	−1.530

### Tat-SID treatment reduces global promoter H3K4 trimethylation

**Figure 3:**
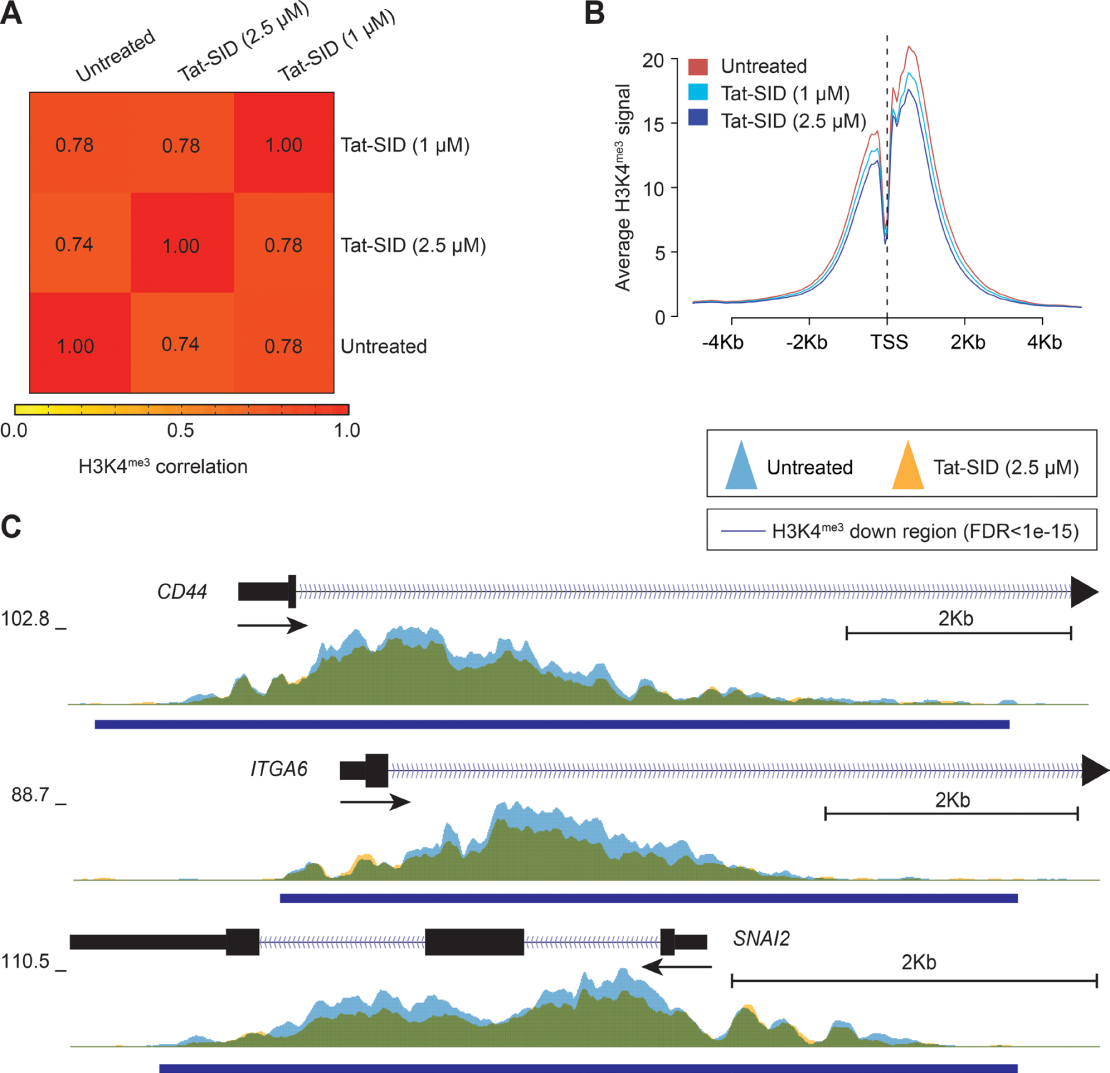
Tat-SID reduces global promoter H3K4 trimethylation **A.** Heatmap representing the correlation (Spearman) of the H3K4^me3^ ChIP signal between untreated (UT), 1 μM Tat-SID treated and 2.5 μM Tat-SID treated MDA-MB-231 cells. Unsupervised hierarchical clustering of samples is shown. **B.** Average H3K4^me3^ ChIP signal at all annotated TSS (−5Kb to +5Kb) in untreated and Tat-SID treated MDA-MB-231 cells. **C.** Overlaid H3K4^me3^ ChIP signal (fold enrichment over input) at the TSS of *CD44, ITGA6* and *SNAI2* in untreated (light blue) and 2.5 μM Tat-SID treated (orange) MDA-MB-231 cells. Regions with significantly decreased H3K4^me3^ signal (FDR < 1 × 10^−15^) are underscored (dark blue bars). Overlapped regions are shown as green.

To better understand the effect of Tat-SID on the epigenetic landscape of TNBC, we performed ChIP coupled with next-generation sequencing (ChIP-Seq) on H3K4^me3^ in MDA-MB-231 cells treated with Tat-SID (1 μM and 2.5 μM). Instead of an expected increase in H3K4^me3^ we observed dose-dependent reduction in H3K4^me3^ at fewer than 10% of the genome-wide transcription start sites (TSS) of annotated genes (Figures 3A, 3B, S5A and Table S7). Analysis of the ChIP-Seq data using SICER-df and Bedtools identified 124 (1 μM Tat-SID) or 2313 gene promoters (2.5 μM Tat-SID) with significant promoter H3K4^me3^ reduction (FDR < 1 × 10-15), in contrast to relatively few genes with H3K4^me3^ increase (FDR < 1 × 10-15) (Figure S5A and Table S7). Given that Tat-SID treatment perturbed the SIN3A-KDM5B interaction, we performed a comparison of Tat-SID target gene promoters with those known to be KDM5B targets [30]. Interestingly, we found that promoters with reduced H3K4^me3^ after treatment with Tat-SID were significantly enriched for KDM5B binding (*p* < 0.0001) (Figure S5B and S5C). These promoters included *CD44*, *ITGA6* (CD49f) and *SNAI2* (SLUG) that are known to regulate the mammary gland stem cell state (Figure 3C) [32]. ChIP-Seq analysis with Tat-SID peptide did not indicate significant epigenetic remodeling of H3K4 trimethylation at the *CDH1* and *ESR1* promoters as found in our previous study [15]. However, in that study, SID peptide was expressed from a plasmid vector over a longer time period.

### Tat-SID impairs invasive morphogenesis and induces anti-tumor effects

**Figure 4:**
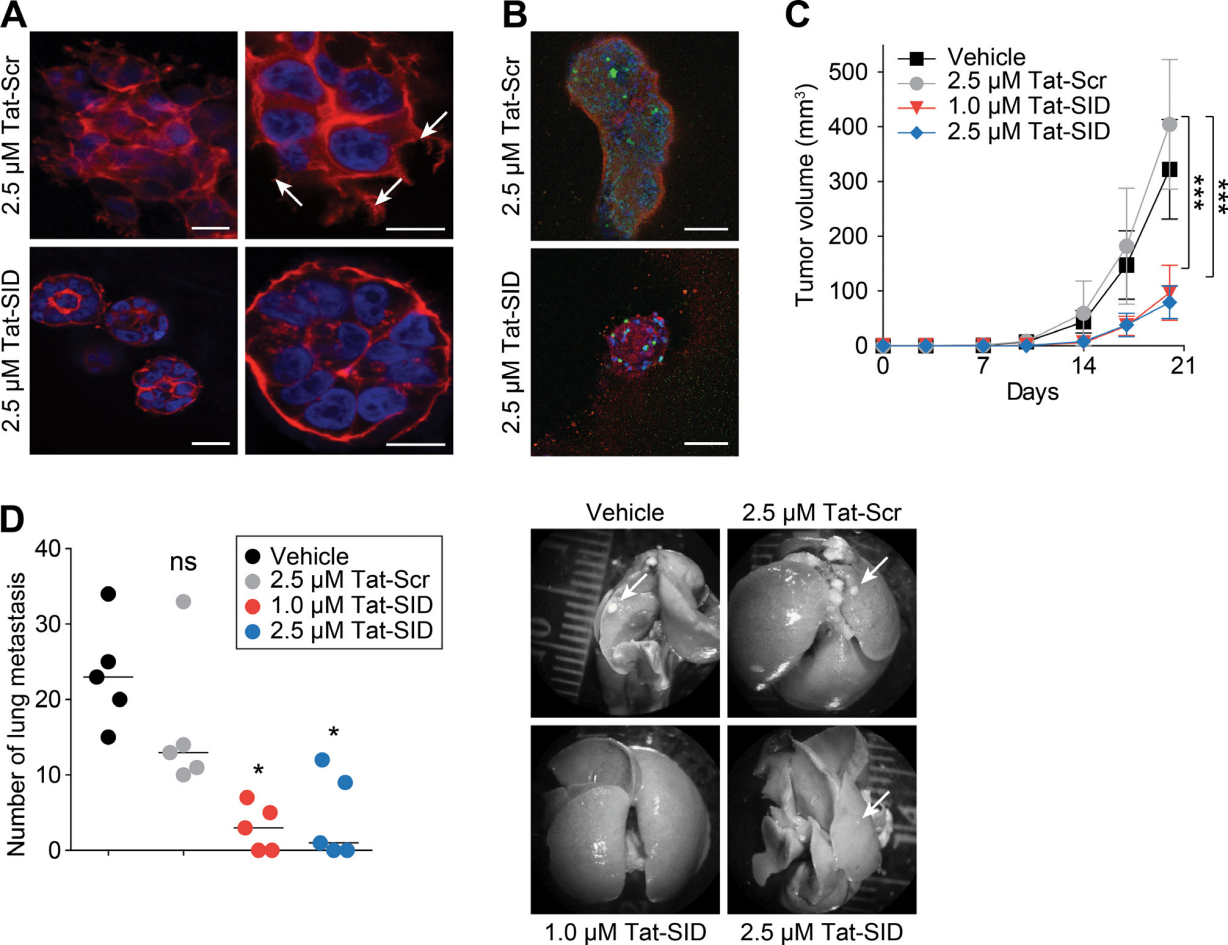
Tat-SID impairs invasive morphogenesis and induces anti-tumor effects **A.** Colony morphogenesis of MDA-MB-231 cells treated with Tat-Scr or Tat-SID at the indicated concentrations. Cells were cultured in 3D matrigel for 10 days followed by staining of colonies with phalloidin (red) and DAPI (blue nuclear stain). Arrows indicate invasive projections. Scale bars: 25 μm (left), 10 μm (right). **B.** MDA-MB-231 cells were treated with Tat-Scr or Tat-SID and cultured as described in (A). Colonies were stained with caspase-3 (green) and E-cadherin (red). Scale bars: 50 μm. **C.** Tumor progression in Balb/c mice (*n* = 5) inoculated with 4T1 cells treated with water, Tat-Scr (2.5 μM) or Tat-SID (1 μM and 2.5 μM) for 14 days, and tumor volume quantified at the indicated time points. Tat-Scr versus 1 μM Tat-SID day 20, ***, *p* = 0.0007; Tat-Scr versus 2.5 μM Tat-SID day 20, ***, *p* = 0.0003; p, unpaired *t*-test. **D.** Lungs from sacrificed animals in (C) (n = 5) were isolated and quantified for the number of metastasis observed. Left panel is quantification of number of metastatic foci observed on the surface of the lungs. Right panel is the image of lungs isolated from the sacrificed mice. Tat-Scr versus untreated, ns, *p* = 0.2107; Tat-Scr versus 1 μM Tat-SID, *, *p* = 0.0185; Tat-Scr versus 2.5 μM Tat-SID day 20, *, *p* = 0.0446; p, unpaired *t*-test. Error bars represent mean ± SD.

Tat-SID treatment of MDA-MB-231 3D cultures in basement membrane matrix that closely mimics the tumor microenvironment exerted a strong anti-invasive effect (Figure 4A) characterized by the presence of small (50-100 μM diameter), non-invasive spherically organized colonies in contrast to the large (>200 μM average diameter) disorganized colonies with invasive projections observed with Tat-Scr control (Figure 4A). Although Tat-SID treated colonies resembled acini-like spheroids with increased levels of E-cadherin and cleaved caspase-3, no evidence of full cavitation or mature lumen formation was found (Figure 4B). The loss of invasive potential we observed *in vitro* was reproduced *in vivo* using 4T1 cells, which closely mimic tumor growth and metastatic spread of stage IV human breast cancer in BALB/c. 4T1 cells were treated *ex vivo* for 14 days with Tat-SID, which resulted in no significant change in cell numbers compared to Tat-Scr control. However, when equal numbers of these cells were inoculated orthotopically as allografts into the inguinal mammary gland number 4 of BALB/c female mice, Tat-SID treated cells generated tumors that grew significantly slower than controls, resulting in a 4.2-fold reduction in mean tumor volume and 2.3-fold reduction in mean tumor mass after 20 days (Figures 4C and S6A). *Ex vivo* Tat-SID treatment of 4T1 cells also led to a dramatic reduction in the number and size of lung metastasis (a median value of 3 for 1 μM Tat-SID; 1 for 2.5 μM Tat-SID versus 23 for vehicle and 13 for Tat-Scr) (Figure 4D). In comparison to vehicle treated, decrease in lung metastasis was observed with Tat-Scr but it was not statistically significant. Similarly, tumor growth of MMTV-Myc cells treated *ex vivo* with Tat-SID was found to be impaired 2.1-fold 12 days after injection (Figure S6B).

### Blocking SIN3-PAH2 interactions reduces tumor-initiating TNBC stem cells

**Figure 5:**
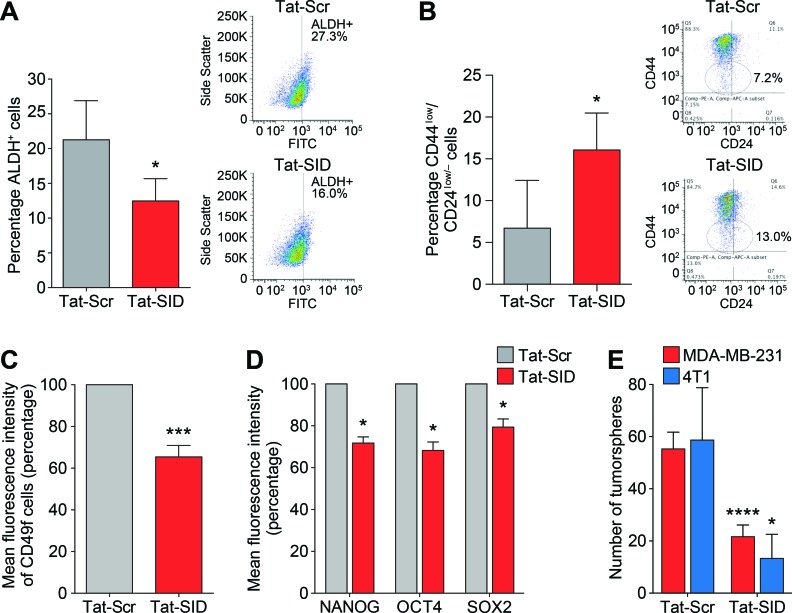
Tat-SID induces decreased expression and activity of CSC markers in TNBC cells **A.** ALDH1 activity in MDA-MB-231 cells treated with 1 μM Tat-Scr (grey) or Tat-SID (red) for 72 h. Results are quantified as percentage of ALDH+ cells. Left panel is a graph showing the mean ALDH activity from three independent experiments. Right panel is plot from one representative experiment. *, *p* = 0.0395; p, unpaired *t*-test. **B.** FACS analysis showing changes in the CD44^low^/CD24^low/neg^ population in MDA-MB-231 cells treated with 1 μM Tat-Scr (grey) or Tat-SID (red) for 72 h. Results are quantified as percentage of cell population. Left panel is a graph showing the mean value from three independent experiments. Right panel is plot from one representative experiment. *, *p* = 0.01; p, unpaired *t*-test. **C.** FACS analysis for CD49f positive cells in MDA-MB231 cells treated with 1 μM Tat-Scr (grey) or Tat-SID (red) for 72 h. Results are quantified as mean fluorescence intensity of CD49f positive cells. ***, *p* = 0.0002; p, one sample *t*-test. **D.** FACS analysis for NANOG, OCT4 and SOX2 staining in MDA-MB-231 cells treated with 1 μM Tat-Scr (grey) or Tat-SID (red) for 72 h. Result is quantified as mean fluorescence intensity from three independent experiments. NANOG, *, *p* = 0.0112; OCT4, *, *p* = 0.0157; SOX2, *, *p* = 0.0345; p, one sample *t*-test. **E.** Tumorsphere assay in MDA-MB-231 (red) and 4T1 cells (blue) treated with 1 μM Tat-Scr or 1 μM Tat-SID for 72 h. Results are quantified for number of tumorspheres. MDA-MB-231, ****, *p* < 0.0001; 4T1, *, *p* = 0.0239; p, unpaired *t*-test. Error bars represent mean ± SD (*n* = 3).

Our results suggested that Tat-SID modulates transcriptional and epigenetic program governing EMT and CSC maintenance in TNBC (Figure 3, Figure 4, Table 1 and Tables S1-S6). We therefore analyzed Tat-SID induced changes in the expression of established CSC markers as defined by increased ALDH activity and a CD44^high^/CD24^low/neg^ antigenic state [33-35]. Basal-B sub-type cell lines such as MDA-MB-231 have increased ALDH activity and display a CD44^high^/CD24^low/neg^ antigen profile [36-38]. Tat-SID treatment significantly reduced the ALDH activity (12.5% ALDH+ cells versus 21.3% ALDH+ cells in controls, Figure 5A). Similar results were also obtained in mouse 4T1 cells (Figure S7). Tat-SID also altered the ratios of CD44 and CD24 double-positive cells, leading to an increase in cell populations defined by CD44^low^/CD24^low/neg^ (16.0% versus 6.7% in controls, Figure 5B). Levels of another important breast CSC marker, CD49f [39, 40], were also downregulated (36% reduction, Figure 5C). Expression of NANOG, SOX2 and OCT4 proteins, hallmarks of stem cell pluripotency and self-renewal, were also downregulated in MDA-MB-231 cells treated with Tat-SID (Figure 5D). This reduction in stem cell markers correlated with significantly impaired growth and a 2.5-fold reduction in tumorsphere formation (Figure 5E). Similarly, the number of mouse 4T1 tumorspheres was reduced 4.5-fold in response to Tat-SID (Figure 5E).

### PF1 modulates the stem-like traits of tumor-initiating CSCs

**Figure 6:**
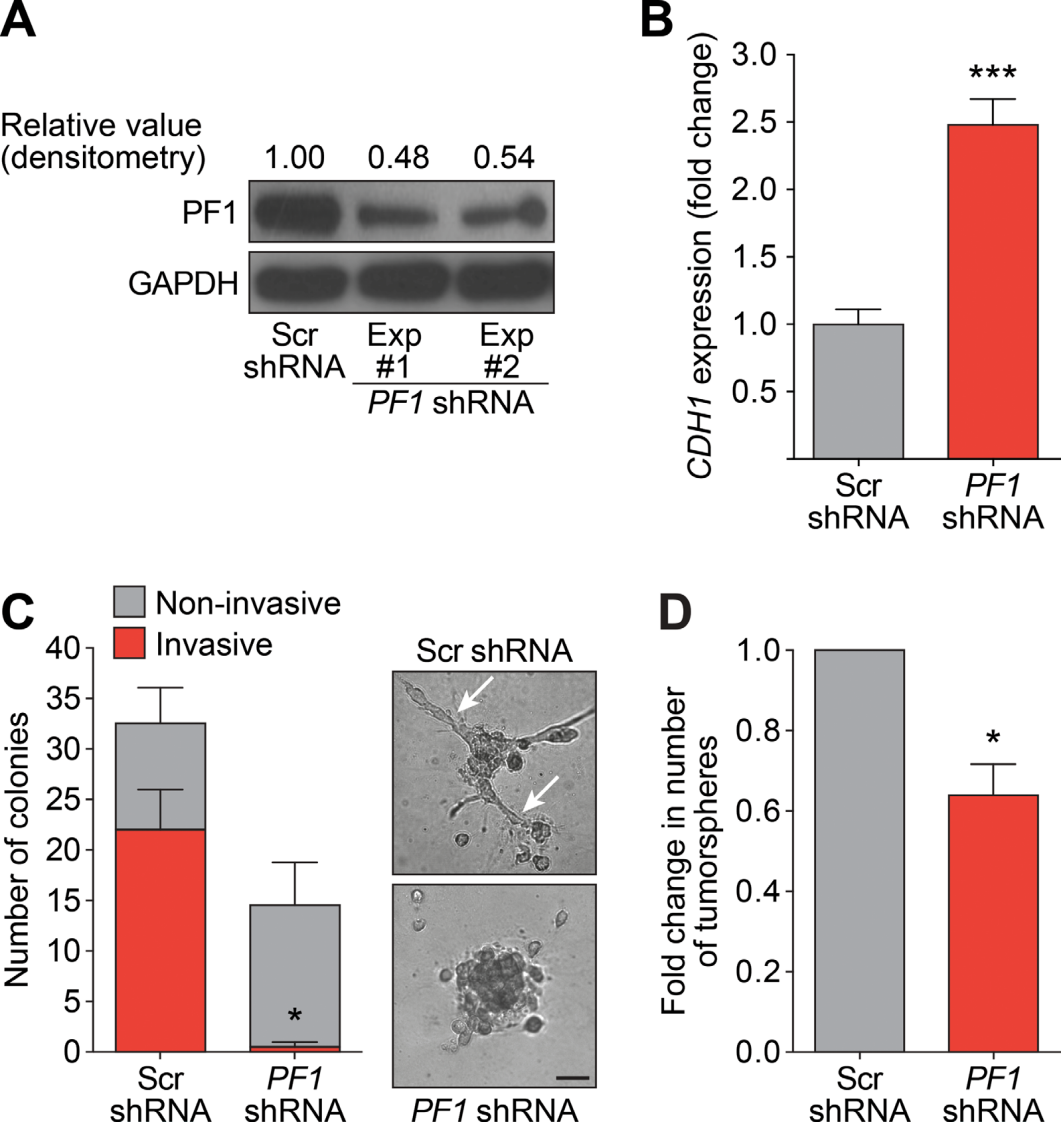
*PF1* Knockdown inhibits the formation of invasive colonies and tumorspheres **A.** Immunoblot analysis of PF1 expression in MDA-MB-231 cells in two independent PF1-shRNA transfection experiments (Lane 2 and 3) in comparison to Scr-shRNA transfection (Lane 1). Numbers represent densitometric analysis of blotted protein bands. **B.** qRT-PCR for expression of *CDH1* mRNA in MDA-MB-231 cells stably transfected with Scr-shRNA (grey) or *PF1*-shRNA (red). ***, *p* = 0.0003; p, unpaired *t*-test. **C.** Left, quantification of the invasive (red) and non-invasive colonies (grey) formed by MDA-MB-231 cells stably transfected with Scr-shRNA or *PF1*-shRNA and cultured on basement membrane extract. Right, phase contrast images of the colonies formed. The arrows point to invasive projections typical of an invasive colony. Error bars represent mean ± SD (*p* = 2). Invasive cells, *, *p* = 0.0334; p, unpaired *t*-test. Scale bar: 100 μm. **D.** Tumorsphere assay for PF1-shRNA (red) and Scr-shRNA (grey) transfected MDA-MB-231 cells. *, *p* = 0.015; p, one sample *t*-test, (*n* = 3). Error bars represent mean ± SD.

**Figure 7:**
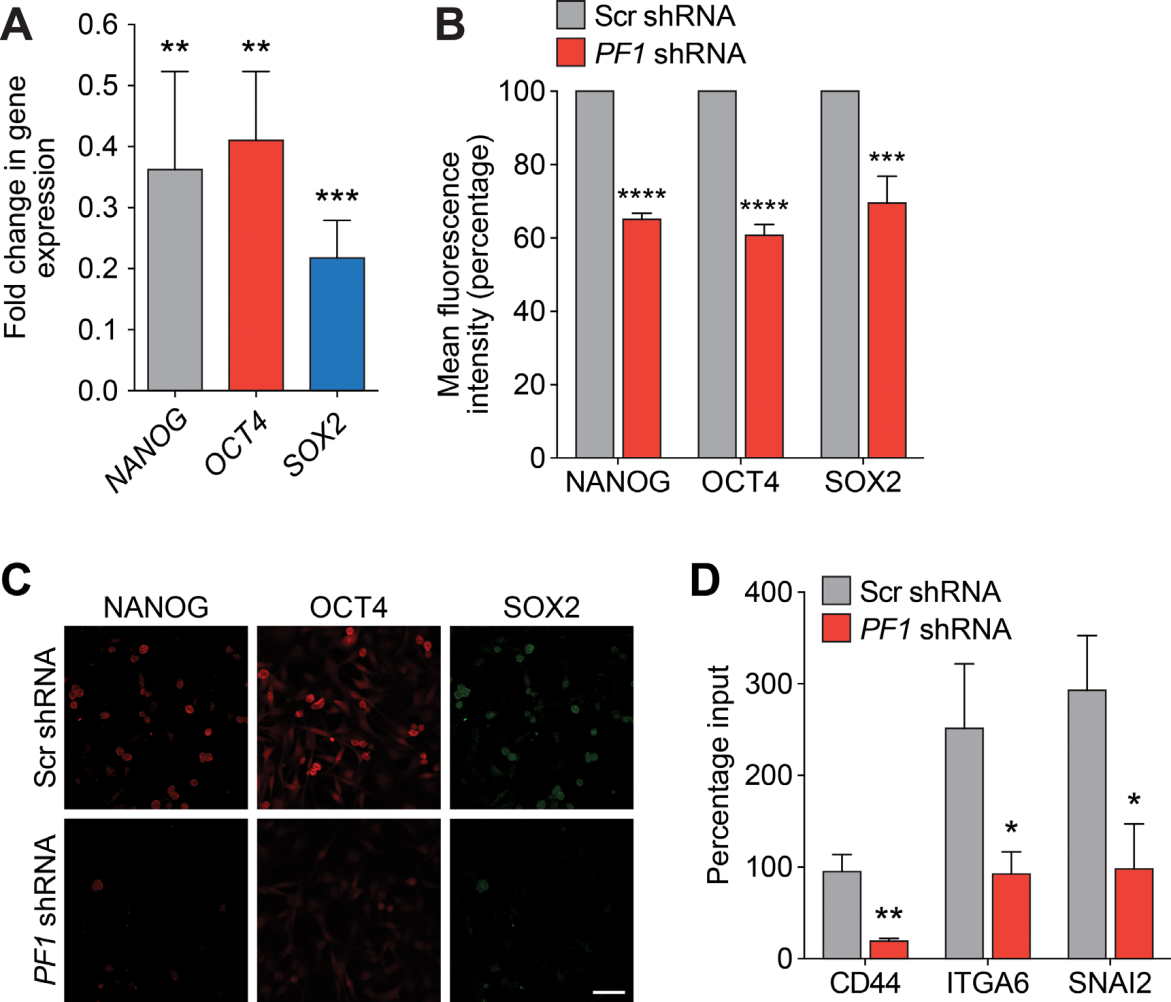
PF1 regulates the expression of TNBC stem cell genes **A.** qRT-PCR for expression of *NANOG* (grey), *OCT4* (red) and *SOX2* (blue) in MDA-MB-231 cells transfected with PF1-shRNA relative to Scr-shRNA control. *NANOG*, **, *p* = 0.0042; *OCT4*, **, *p* = 0.0019; *SOX2*, ***, *p* = 0.0001; p, unpaired *t*-test, (n = 3). **B.** FACS analysis of NANOG, OCT4 and SOX2 proteins in MDA-MB-231 cells transfected with Scr-shRNA (grey) or PF1-shRNA (red). Results are quantified as mean fluorescence intensity from three independent experiments. Error bars represent mean ± SD (*n* = 3). **C.** Confocal images of MDA-MB-231 cells stably transfected with Scr-shRNA or PF1-shRNA and stained with antibodies against NANOG (red), OCT4 (red) and SOX2 (green). Scale bar: 50 μm. **D.** ChIP analysis of *CD44, ITGA6* and *SNAI2* gene promoters for H3K4^me3^ in MDA-MB-231 cells transfected with Scr-shRNA (grey) or *PF1*-shRNA (red). *CD44*, **, *p* = 0.0023; *ITGA6*, *, *p* = 0.0205; *SNAI2*, *, *p* = 0.0120; p, unpaired *t*-test, (*n* = 3). Error bars represent mean ± SD.

Recent research has revealed that PF1 is highly expressed during chick neural crest EMT, recruiting Snail2 and HDACs to specifically repress transcription of the adhesion molecule Cad6b (Cadherin6b) and E-cadherin [41]. Given that Tat-SID disrupted the binding between PAH2 domain of SIN3A and PF1 (Figure 1), we further investigated the role of PF1 function in modulation of EMT and CSC. MDA-MB-231 cells were stably transfected with *PF1*-shRNA or non-specific scrambled (Scr) shRNA (Figure 6A). Consistent with a role for PF1 in the regulation of *CDH1* expression, a 2.5-fold increase in *CDH1* was observed after *PF1* knockdown (Figure 6B). Further supporting our finding that suggests disruption of the SIN3A-PF1 interaction underpins the molecular and phenotypic changes observed with Tat-SID, we observed a 2-fold reduction in 3D colony-forming potential and a 20-fold reduction (3.4% versus 67.7%) in invasive colonies in cells transfected with PF1 shRNA compared to control (Figure 6C). *PF1* knockdown was also accompanied by a 1.5-fold reduction in the tumorsphere-forming ability of MDA-MB-231 cells (Figure 6D). Also consistent with Tat-SID treatment, PF1 depletion significantly reduced mRNA and protein levels of NANOG, OCT4 and SOX2 (Figure 7A-7C). Consistent with our ChIP-Seq results, knockdown of PF1 in MDA-MB-231 cells resulted in 5-fold, 2.7-fold and 3-fold reduction, respectively, in H3K4^me3^ enrichment at the *CD44*, *ITGA6* and *SNAI2* promoters (Figure 7D).

**Figure 8:**
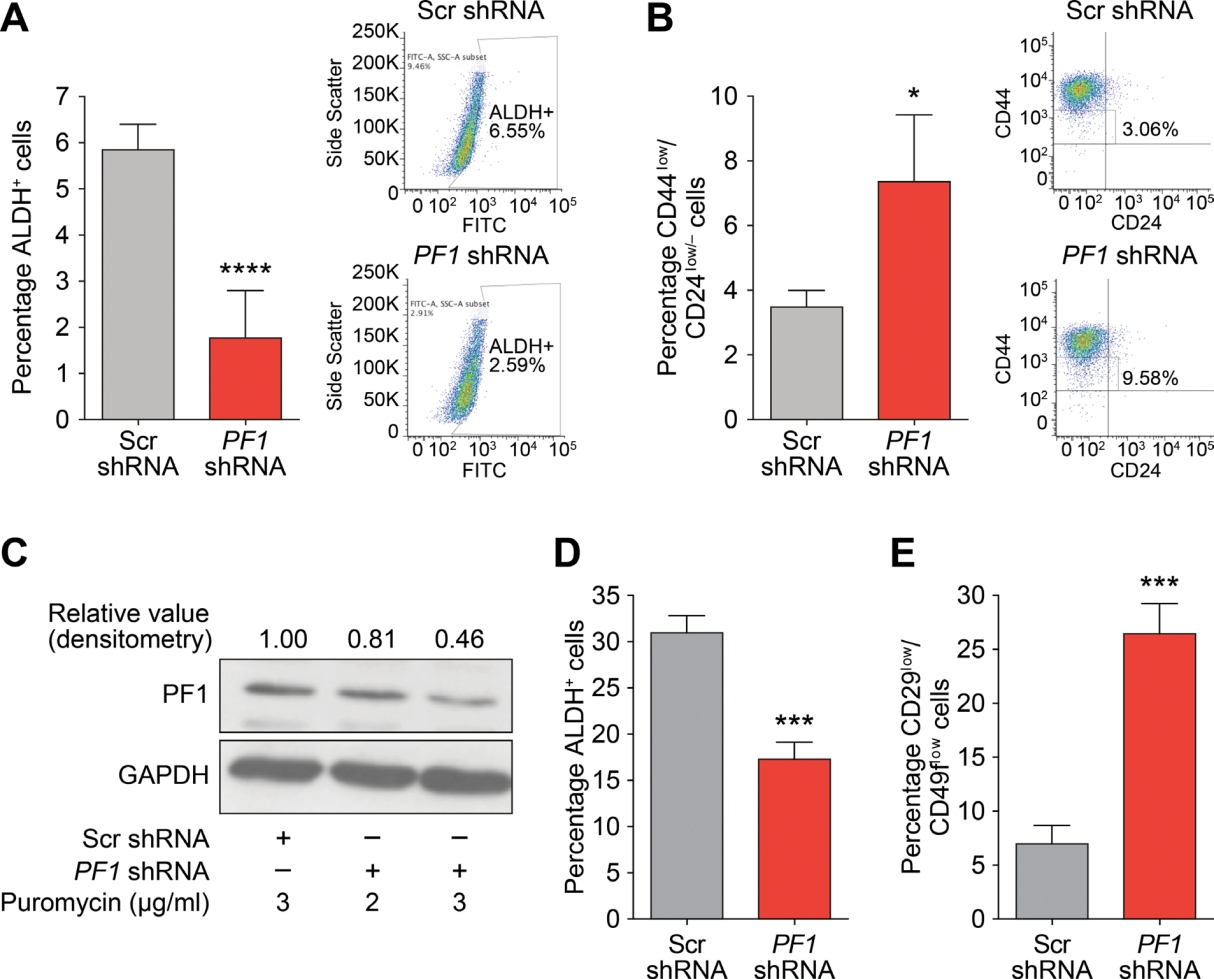
PF1 maintains a stem cell phenotype in TNBC **A.** ALDH1 activity in MDA-MB-231 cells transfected with Scr (grey) or PF1-shRNA (red). Results are quantified as percentage of ALDH+ cells. Left panel is graph showing the mean value from three independent experiments and the right panel is plot from one representative experiment. ****, *p* < 0.0001; p, unpaired *t*-test, (*n* = 3). **B.** FACS analysis showing changes in the CD44^low^/CD24^low/neg^ population in MDA-MB-231 cells transfected with Scr-shRNA (grey) or *PF1*-shRNA (red). Results are quantified based on the percentage of cells. Left panel is graph showing the mean value from three independent experiments and the right panel is plot from one representative experiment. *, *p* = 0.0110; p, unpaired *t*-test, (*n* = 3). **C.** Immunoblot analysis of PF1 expression in 4T1 cells stably transfected with Scr-shRNA or PF1-shRNA and selected with 2 (Lane 2) or 3 μg/ml (Lane 3), of puromycin. Numbers represent the densitometric analysis of the blot. Cells selected with 3 μg/ml puromycin (Lane 3) were used for further experiments. **D.** ALDH1 activity in 4T1 cells transfected with Scr (grey) or PF1 shRNA (red). Results are quantified as percentage of ALDH+ cells. ***, *p* = 0.0008; p, unpaired *t*-test, (*n* = 3). **E.** FACS analysis showing changes in the CD29^low^/CD49f^low/neg^ population in 4T1 cells transfected with Scr (grey) or PF1 shRNA (red). Results are quantified based on the percentage of cells. ***, *p* = 0.0008; p, unpaired *t*-test, (*n* = 3). Error bars represent mean ± SD.

**Figure 9:**
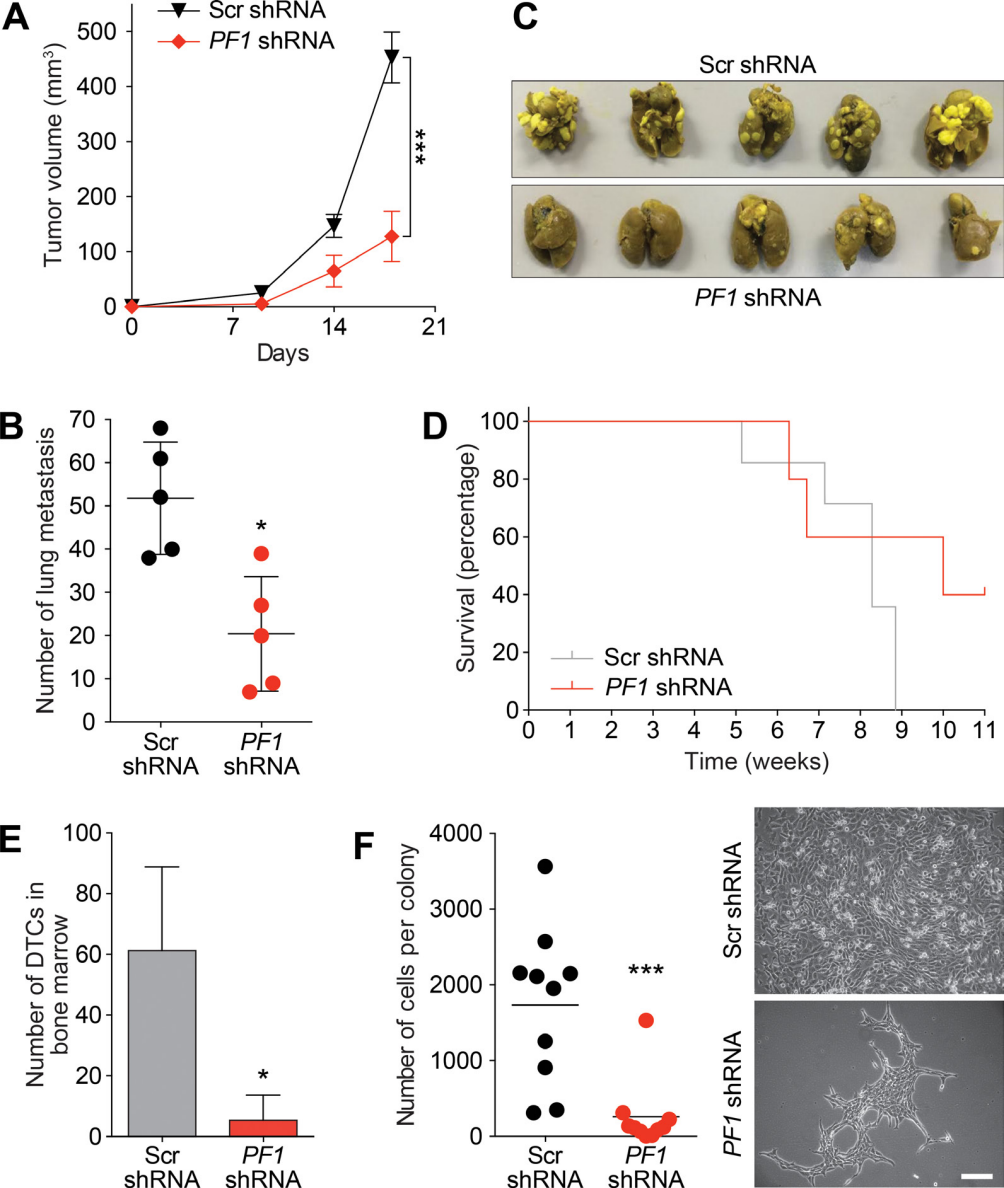
PF1 knockdown inhibits primary tumor growth and disseminated metastatic disease *in vivo* **A.** Tumor progression in Balb/c mice (*n* = 10) inoculated with 4T1 cells transfected with Scr-shRNA (black) or *PF1*-shRNA (red). Tumor volumes were quantitated at the indicated time points. Day 18, ***, *p* = 0.0001; p, unpaired *t*-test. Primary tumors were resected, and after 35 days five mice were sacrificed for analysis of lung metastasis (described in panel B). The remaining five mice were analysed for survival (panel D) and the presence of disseminated tumor cells (DTCs) in bone marrow (panel E and F). **B.** Lungs from sacrificed Balb/c mice inoculated as described in (A) were isolated and quantified for the number of metastasis observed. *, *p* = 0.0159; p, unpaired *t*-test, (*n* = 5). **C.** Images of lung metastasis observed in Balb/c mice sacrificed and isolated in (B). **D.** Kaplan-Meier analysis following removal of primary tumors in Balb/c mice from (A) (*n* = 5). **E.** Quantification of the disseminated 4T1 tumor cells isolated from the bone marrow of sacrificed animals from (D). *, *p* = 0.0139; p, unpaired *t*-test. **F.** Left, graph showing the average number of cells per colony formed by disseminated 4T1 tumor cells from bone marrow of Balb/c mice from (D). ***, *p* = 0.0006; p, unpaired *t*-test. Right, phase contrast images of the colonies formed by the disseminated tumor cells isolated from the bone marrow of mice from (D). Scale bar: 100 μm. Error bars represent mean ± SD.

Supporting a role for PF1 in cancer stem cell maintenance and in agreement with Tat-SID data, PF1 knockdown resulted in a 2.5-fold decrease in ALDH1 positive cells (6.55% in Scr-shRNA versus 2.59% in *PF1*-shRNA; Figure 8A). Moreover, the CD44^low^/CD24^low/neg^ population was enriched 3-fold in cells transfected with PF1 shRNA compared with control (Figure 8B). Similarly, using a different shRNA construct, PF1 knockdown in mouse 4T1 cells (Figure 8C) led to fewer ALDH+ cells (Figure 8D) as well as increase in the proportion of cells with decreased expression of the breast cancer stem cell markers CD49f and CD29 (Figure 8E). *In vivo*, PF1 knockdown in 4T1 cells generated tumors that grew significantly slower than scrambled control, resulting in a 3.5-fold reduction in mean tumor volume after 18 days (Figure 9A). We also found that knockdown of PF1 in 4T1 cells resulted in a significant reduction in the number and size of lung metastasis 35 days after tumor removal (PF1 shRNA, median = 20 versus Scr shRNA, median = 52) (Figure 9B and 9C). Furthermore, mice bearing PF1 knockdown tumors displayed longer overall tumor-free survival compared to controls following tumor excision (Figure 9D). Despite the small sample size (*n* = 5), two mice in which PF1 knockdown tumors were excised showed no clinical disease symptoms and macroscopic lung metastases were not evident when these mice were electively sacrificed. Lastly, we performed an examination of the bone marrow for disseminated tumor cells (DTCs) that are associated with poor outcome in patients with metastatic breast cancer [42, 43]. In agreement with our prior work [15, 16] and results with activity of Tat-SID against dissemination of lung metastases, PF1 depletion in 4T1 cells also led to a significant 12-fold decrease in the number of bone marrow DTCs compared to control (Figure 9E), with DTCs isolated from mice bearing PF1 knockdown tumors proliferating at a slower rate and with 6.7-fold fewer cells per colony (Figure 9F).

## DISCUSSION

In this study we show that Tat-SID disrupts interaction between the PAH2 domain of SIN3 and the PF1 chromatin regulator that is expressed from a locus amplified in breast cancer [17, 18]. Our results strongly suggest that this mechanism underlies the molecular and phenotypic effects arising from treatment with SID peptide, and this also applies to recently described small molecule mimetics of SID (avermectins) [16]. The prior identification of a complex containing chromatin-modifying proteins, PF1, MRG15, EMSY and LID/KDM5A, that was found to interact with SIN3 [20, 23-25, 44] led us to speculate that disruption of histone H3K4 demethylase recruitment could be responsible for the dramatic increases in H3K4^me3^ at the *CDH1* and *ESR1* promoters we observed previously upon exposure to SID peptide [15]. Recent reports suggesting a role for KDM5B in regulation of the EMT program in cancer stem cells [45, 46] and interaction of KDM5B with EMSY [29], prompted us to investigate the SIN3A-KDM5B interaction.

While our finding that 3-days Tat-SID treatment led to a decrease in genome-wide H3K4^me3^ is in contrast to previously-reported increases in H3K4^me3^ at the *CDH1* and *ESR1* promoters [15], it should be noted that the previous results were observed after a longer time period [15]. This suggests that re-expression of these genes precedes a large increase in H3K4^me3^, which may serve to “lock in” a permissive epigenetic state in response to SID treatment. Thus an increase in histone acetylation through prevention of recruitment of a deacetylase-containing complex may be the initial route for epigenetic remodeling in response to inhibition of PAH2 interactions. Our finding that H3K4^me3^ decreases in response to Tat-SID-mediated disruption of KDM5B is in agreement with recent studies in which KDM5B has been knocked down in embryonic stem cells [47] or breast cancer cell lines [30, 48], as well as in a mouse knockout model [49]. The mechanisms underpinning these results remain to be established but possibilities include a role for KDM5B in fine-tuning epigenetic regulation of genes. Furthermore, the effect on individual genes of blocking interactions between SIN3 and KDM5B may be difficult to predict given that recent research has demonstrated that ‘co-repressor complexes’ including SIN3 can function in transcriptional activation as well as repression [50]. This may also be the case with KDM5B as its *Drosophila* homolog, Lid, has been shown to activate transcription by inhibiting the histone deacetylase activity of Rpd3 in PF1-MRG15 complex [51]. Further characterization of the consequences of PAH2 inhibition should also focus on whether this impacts function of the breast cancer oncoprotein EMSY, which has been shown to interact with PF1 and, more recently, KDM5B to repress expression of the anti-metastatic microRNA miR-31 [25, 29].

The most important finding of this study is the identification of PF1 as a therapeutically targetable factor required for maintenance of EMT and the CSC phenotype. Treatment with both SID peptide and avermectins [16] targets multiple key genes in the EMT pathway, including *TGFB1*, and it is noteworthy that inhibition of TGFβ activity has been associated with loss of KDM5B in basal-like breast cancer cells [30]. Another PAH2-interacting protein, TIEG1 has also been shown to play a role in the TGFβ/SMAD signal transduction pathway [52, 53] and disruption of this interaction also warrants investigation. Passage through EMT contributes to generation and maintenance of tumor-initiating CSCs [5] and genome-wide transcriptional profiling of several breast cancer cell lines has uncovered a relationship between EMT and breast CSCs (BCSCs) [33, 54, 55]. Here, basal-B subgroup cell lines (such as MDA-MB-231 used in this study) were found to express an EMT signature and are thus enriched with cells that have undergone at least a partial EMT and acquisition of CSC properties such as expression of mesenchymal genes and enhanced invasiveness. These include cells with increased ALDH activity and CD44^high^/CD24^low/neg^ antigenic state [33-35]. We observed that both Tat-SID treatment and PF1 knockdown decreased ALDH activity and also shifted the population towards a CD44^low^/CD24+ composition that is associated with a luminal phenotype [33, 55-57]. Our results also show down-regulation of other breast CSC-associated genes/markers like CD49f, ALDH, NANOG, OCT4, and SOX2 in both Tat-SID treated and PF1 knockdown cells. Of these, the promoters of CD49f and CD44 also showed a decrease in the H3K4^me3^ mark. Thus, our results have identified PF1 as a critical factor for the breast CSC phenotype, which is an important step forward in understanding the mechanisms responsible for the maintenance of this cell population. The recent finding that PF1 is highly expressed during chick neural crest EMT, recruiting Snail2 and HDACs to specifically repress transcription of the adhesion molecule Cad6b (Cadherin6b) and E-cadherin [41], strongly suggests that its biological activities in TNBC be investigated further. Expression of SNAIL is an indicator of poor prognosis in breast cancer. This is linked to repression of *CDH1* and induction of EMT that occurs through recruitment of SIN3A and HDACs by SNAIL to E-boxes contained in the CDH1 promoter [59-61]. Whether SNAIL also recruits a SIN3A-PF1-MRG15-KDM5B complex to repress *CDH1* expression and induce EMT remains to be established.

Although the SIN3A PAH2-interacting SID of PF1 possesses structural and sequence homology with MAD SID, the interaction is 10-fold lower in affinity compared with the prototypic SIN3-MAD interaction [31]. Therefore, the use of small molecules based on *in silico* modeling of the MAD-SID sequence to prevent recruitment of the PF1-containing complexes represents a new and potentially clinically effective therapeutic strategy. While it cannot be ruled out that the effects of SID treatment act through inhibiting the interaction of additional PAH2-binding proteins with SIN3, the finding that PF1 knockdown phenocopied Tat-SID suggests that it is the principal target. In light of this, it will be important to determine whether promoters that are epigenetically modulated by PF1 depletion are direct or downstream targets. The number of partner proteins thus far identified for PF1 is relatively small (http://thebiogrid.org) but the identification of retinoblastoma binding protein 7 (RBBP7) and BRCA1 suggest additional potential roles for PF1 including in DNA repair.

Our results strongly point to gene- and pathway-specific modulation of epigenetic markers and transcription in response to Tat-SID. This results in dramatic *in vitro* phenotypic changes characterized by partial differentiation, reversal of EMT and decreased CSCs that translate into significantly reduced metastatic disease dissemination *in vivo*. Selective inhibition of SIN3A function using SID decoy leads to clinically relevant epigenetic reprogramming in TNBC and defines the SIN3A-PF1 protein interaction as a *bone fide* therapeutic target.

## MATERIALS AND METHODS

### Cell culture, plasmids and transfections

The mouse metastatic mammary 4T1 tumor cell line (Cat# CRL-2539) and human MDA-MB-231 breast adenocarcinoma cell line (Cat# HTB-26) were purchased from the American Type Culture Collection (ATCC). The mouse mammary tumor MMTV-Myc cell line has been previously reported [62, 63]. Cell lines were authenticated by short tandem repeat (STR) profiling in accordance with the standard ASN-0002-2011 in April 2015 (DDC Medical). 4T1 cells were maintained in RPMI supplemented with 10% fetal bovine serum (FBS) and 1% Antibiotic-Antimycotic solution (Invitrogen). The MDA-MB-231 cell line was maintained in DMEM supplemented with 10% FBS, 1% GlutaMAX (Invitrogen), 10mM HEPES, 1mM sodium pyruvate, non-essential amino acids and 1% antibiotic-antimycotic solution. MMTV-Myc cells were cultured in DMEM/F12 medium supplemented with 5% FBS, 1% GlutaMAX, 10mM HEPES, and 1% antibiotic-antimycotic solution. Stable knockdown of PF1 in MDA-MB-231 was performed with pLKO-PF1 shRNA (clone# TRCN0000015704), which was a kind gift from the laboratory of Gregory David (NYU School of Medicine, NY, USA) [44]. A second pLKO-PF1 shRNA construct (clone# TRCN0000084422, Sigma Aldrich) was used for stable knockdown in mouse 4T1 cells. Stable transfections were performed with 1 μg of DNA using TurboFect (ThermoScientific) according to the manufacturer's recommendations.

### Peptides

MAD-SID peptide (Tat-SID: YGRKKRRQGGG-VRMNIQMLLEAADYLERRER) with or without FITC conjugation, MAD scrambled peptide (Tat-Scr: YGRKKRRQGGG-EQRARRIMERLLEYNMVADL), PF1-SID (YGRKKRRQGGGQLRRPFELLIAAAMERNPTQ) and PF1 scrambled (YGRKKRRQGGGRLFMQLELRATPAEAPINQR) were synthesized to a purity level of 95% as assessed by analytical reversed phase-high performance liquid chromatography (BioSynthesis, Inc).

### Peptide internalization assay

Sub-confluent cultures of MDA-MB-231 cells were treated with FITC-conjugated Tat-SID (1 μM) for 2 h and 24 h. For flow cytometry the cells were trypsinized and resuspended in 1% BSA-PBS solution and analyzed using flow cytometer BDCanto (BD BioSciences). For confocal imaging cells were washed with PBS and mounted using Prolonged Gold Antifade with DAPI (Molecular Probes) and analyzed using Leica SP5 confocal microscope.

### Purification of the PAH2 domain of SIN3A

The PAH2 domain of mSin3A was overexpressed in the *E. coli* BL21 (DE3) codon plus RIL strain (Stratagene) by addition of 1 mM isopropyl-1-thio-D-galactopyranoside and incubation overnight at 15°C. Harvested cells were re-suspended in 50 mM sodium phosphate buffer, pH 7.4, supplemented with 500 mM sodium chloride, 5% glycerol, and 0.1% Igepal CA-630 and lysed using a microfluidizer (Micro-fluidics) at 20,000 psi. After clarification of the crude extract by high-speed centrifugation, the lysate was loaded onto a 5 ml HiTrap chelating column (GE Healthcare) charged with Ni2+. The column was washed and the protein was eluted with 30 mM HEPES pH 7.4, 250 mM sodium chloride, and 250 mM imidazole. The protein was next purified on a Superdex75 column (GE Healthcare) equilibrated with 20 mM Tris-HCl buffer, pH 8.0, and 150 mM sodium chloride. Fractions containing the pure protein were combined and concentrated with 3 kDa MWCO centrifugal filters (Amicon).

### Competition assay for pSID peptide binding affinity

The binding affinity of pSID for SIN3A was assessed in a fluorescence anisotropy competition assay using a fluorescein isothiocyanate-labeled Mad1 peptide as an assay probe. Competition experiments were performed with 70 nM purified mSin3A PAH2 domain and 10 nM fluorescent probe and increasing concentrations of unlabeled competing pSID in a PBS buffer (pH 7.4) with 0.01% BSA in total volume of 40 μL. Measurements were obtained after a 1 h incubation of the fluorescent ligand and the protein at 25°C with a Safire 2 microplate reader (Tecan). Assuming a one-site competitive binding model, the data was fit using Prism software.

### Immunofluorescence

MDA-MB-231 cells were cultured on 8 chambered wells (BD Biosciences) and fixed with 4% paraformaldehyde/PBS for 15 min at room temperature. For 3D cultures cells were seeded (3 × 10^3^/well) in quadruplicate onto Matrigel (BD Biosciences) or Cultrex basement membrane extracts (Trevigen) in 8-well culture slides to prepare three-dimensional cultures as described earlier [64]. The media was changed every 48 h for 8 consecutive days. Colony morphology was determined by phase-contrast microscopy. For immunostaining, cells were permeabilized with 0.5% Triton X-100/PBS and blocked with 10% normal goat serum (Invitrogen) in PBS for 1 h. Primary antibodies were incubated overnight at 4°C in blocking buffer and washed 3 times with washing buffer (0.05% Triton X-100/PBS) and once with PBS. Secondary antibodies (dilution 1:200 in 1% normal goat serum/PBS) were added for 1 h and then washed. The samples were then mounted with ProLong Gold antifade reagent with DAPI (Molecular Probes/Invitrogen, CA), following the manufacturer instructions. All incubations and washes were done at 4 or 25°C as required. Confocal microscopy was performed using a Leica SP5 confocal microscope at the Shared Instrumentation facility of department of Hematology at Mount Sinai School of Medicine, NY.

### Proximity ligation assay

MDA-MB-231 cells plated onto coverslips in 12 well plates with or without Tat-SID treatment were stained with monoclonal SIN3A (sc-5299) 1:100 and polyclonal KDM5B (ab50958) 1:1000 following the Duolink protocol according to the manufacturer's instructions (Olink Bioscience) except utilizing 1% BSA in PBS as a blocking reagent and carrying out initial washes in PBS. Cells were counterstained in To-pro-3-iodide in PBS, 3×5 min washes at RT and mounted in Vectashield mounting medium (vector labs). Images were collected on a Zeiss LSM700 confocal microscope and the Duolink software was utilized to quantitate the signals.

### Co-IP

MDA-MB 231 cells were treated with 1 μM Tat-SID peptide. Nuclear Extracts were used for immunoprecipitation (IP) with SIN3A (Abcam, ab3479) and KDM5B (Abcam, ab27689) antibodies using IP kit from Thermo Scientific and probed with SIN3A, KDM5B, PF1 or MRG15 antibody by immunoblot analysis.

### Tumorspheres assay

Between 1 to 5.0 x10^3^ MDA-MB-231 or 4T1 or cells treated with 1μM Tat-SID or Tat-Scr for 72 h, were plated in ultra low adhesion 6-well plates (Corning, Corning, NY) and incubated in serum-free F12/DMEM (Cellgro) supplemented with 20ng/ml EGF, 0.5% Matrigel and 1:50 B27 Supplement (Invitrogen) for 8-10 days at 37°C in a humidified atmosphere of 5% CO2. The number of tumorspheres per well (triplicates) were counted.

### Quantification of cancer stem cell markers

For aldehyde dehydrogenase assay, cells were dissociated with PBS-EDTA and tested for ALDH activity (2 × 10^5^ cells/sample), using the Aldefluor assay (Aldegen) according to the manufacturer's instructions. For CD44 and CD24 antigens, cells were dissociated with Accutase, washed with PBS and incubated with PE-conjugated anti-CD24 and APC-conjugated anti-CD44 antibodies (BD Biosciences) for 40 minutes in ice. For quantification of NANOG, OCT4 and SOX2 dissociated cells were fixed with 1% paraformaldehyde (15 min at RT), permeabilized with 0.5% TritonX100 (10 min at RT) and incubated with 1:100 diluted antibodies against NANOG, OCT4 and SOX2 (Cell Signaling) for 1 h at room temperature. The cells were then washed and incubated with fluorophore-conjugated secondary antibodies Abcam). FACS analysis was carried out using a FACScanto flow cytometer, DIVA software program for acquisition (BD Biosciences) and FlowJo (Treestar.) software for analysis.

### Quantitative real-time PCR

RNA was isolated using RNeasy Plus Mini Kit (Qiagen), and cDNA was prepared using Superscript First-Strand Synthesis System for RT-PCR Kit (Invitrogen) or iTaqScript (Bio-Rad), all following manufacturers’ instructions. Quantitative real-time PCR was performed using manufacturers’ instructions for QuantiTect SYBR Green PCR (Qiagen) or iTaq Universal SYBR Green Supermix (Bio-Rad) kits on Opticon or CFX96 machines (Bio-Rad) with annealing temperature 54°C with 50-250 ng cDNA per reaction. For determination of gene expression the “delta-delta Ct method” was used relatively to RPL30 housekeeping genes. PCR primers are listed in supplementary Table S8.

### Affymetrix expression analysis

Sub-confluent cultures of MDA-MB-231 cells were treated with 1 μM scrambled (Tat-Scr) or SID peptide (Tat-SID) for 24 h. Total RNA was isolated using the ZR RNA MiniPrep Kit (Zymo Research). The concentration and quality of the total RNA was assessed on an Agilent 2100 BioAnalyzer (Agilent Technologies). All samples were normalized to 200ng and processed according to standard Affymetrix protocols using GeneChip WT Terminal Labeling and Controls Kit (Affymetrix) and WT Expression Kit (Ambion). The quality and quantity of labeled cRNA was checked and 750 ng of labeled cRNA were hybridized to a GeneChip Human Gene 1.0 ST Arrays using GeneChip Hybridization, Wash, and Stain Kit (Affymetrix). The arrays were scanned on a GeneChip Scanner 3000 7G. Affymetrix array data were analyzed by Chipinspector 2.1 (Genomatix). Transcripts were considered significantly regulated if at least 3 significant probes mapped to them and the log2 fold change of the transcript calculated from these probes was above 1 or below −1. For all subsequent analyses, we used the median expression values of two independent biological replicates. Replicates were combined exhaustively, i.e. mean fold changes were calculated by comparing each replicate from the treatment group to each replicate from the control group. Log2 fold change values for genes were calculated as the average of the log2 fold change values of the corresponding significantly regulated transcripts and a False Discovery Rate (FDR) was set as 5%. Expression microarray analysis was performed according to Minimum Information About a Microarray Gene Experiment (MIAME) guidelines and data have deposited on the Gene Expression Ontology (GEO) database with the series accession number GSE73278. The GEO superseries accession number for this study is GSE73871.

### Pathway and network analysis

Ingenuity Pathway Analysis (IPA) software (www.ingenuity.com) was used to identify significantly overrepresented pathways, cellular functions and upstream transcription factor analysis in the list of identified proteins. The Tat-SID versus Tat-Scr peptide expression data were imported into IPA and filtered on 2-fold change before a core analysis was performed to identify the most significantly regulated proteins and associated cellular functions.

### ChIP-Seq

Native ChIP-seq for H3K4^me3^ (Abcam, ab1012) was performed in untreated, 1 μM and 2.5 μM Tat-SID treated MDA-MB-231 cells as previously described [65]. Input DNA was used as control for the background. High throughput sequencing on all samples was performed using Illumina HiSeq 2500 with single-end sequencing of 100nt (Mount Sinai Genomic Core Facility). Sequencing reads were quality checked by FastQC (version 0.10.0) and NGS-QC generator (version 1.5.1) [66] prior to analysis. Summary of ChIP QC is shown in Table S9. Sequence reads were then aligned to the Genome Reference Consortium Human Build 37 genome (hg19) with Bowtie (version 1.0.0) [67] using the following parameters: seed length (l) = 70 bp, maximum mismatch (n) = 2, suppression (m) = 20, and reported alignments (k) = 1. MACS2 program (version 2.1.0) [68] was used to generate Bedgraph files that show fold change enrichment of ChIP over input. Bedgraph files were then converted into BigWig files by BedClip program and uploaded onto UCSC genome browser for visualization and plotting. SICER-df program [69] was used to reveal significantly changed peaks between the untreated and Tat-SID treated MDA-MB-231 cells using the following parameters. For H3K4me3: window size = 50bp, gap size = 400bp, island calling = FDR<1×10^−4^, UT versus Tat-SID = FDR<1×10^−8^. Genes with significant histone modification changes were determined by intersecting significantly changed peaks of H3K4me3 ChIP (by SICER-df) to ±3 Kb and ±10 kb TSS of all RefSeq genes, respectively, using Bedtools. Regions and genes with significant ChIP signal changes after Tat-SID treatment are summarized in Table S7. TSS analyses were performed using the SitePro tool from Cistrome (http://cistrome.org) [70]. The bed files containing genomic positions around TSS were generated using RefSeq gene annotation downloaded from UCSC Genome Browser (http://genome.ucsc.edu). Only the longest isoform of each gene was used to prevent double plotting of the same genomic region. Hierarchical clustering and correlation heatmap between each ChIP samples were generated with a 100 bp window and Spearman correlation using bamCorrelate function from deepTools program. Histone modification snapshots were generated using UCSC Genome Browser. Chi-Square test from GraphPad program (http://graphpad.com/quickcalcs/chisquared1.cfm) was used to calculate the p value of KDM5B binding enrichment at H3K4me3 down genes after Tat-SID treatment. ChIP-Seq data have deposited on the Gene Expression Ontology (GEO) database with accession number GSE73869. The GEO superseries accession number for this study is GSE73871.

### Chromatin immunoprecipitation (ChIP)

Native ChIP for H3K4^me3^ (Abcam, ab1012) was performed in MDA-MB-231 cells that were either untreated, treated with 1 μM or 2.5 μM Tat-SID, or transfected with Scr-shRNA or PF1-shRNA as previously described [65]. Input DNA was used as control for the background. DNA obtained from input or immunoprecipitated DNA was analyzed by real-time PCR using primers mapping to the *CD44*, *ITGA6* and *SNAI2* promoter regions. Percent Input method was used for analysis. The following PCR primers were used: CD44-F 5′-TCACATAGCCAGGAGCAGTG-3′; CD44-R 5′-AAATCCTTCCCTCCCTGAAA-3′; ITGA6-F 5′-ACCTCCCAGGAGAAAGAGGA-3′; ITGA6-R 5′-GCGACTAAGCGCCAAAATAC-3′, SNAI2-F 5′-CAGACCCTGGTTGCTTCAA-3′; SNAI2-R 5′-CTTCATGCAAATCCAACAGC-3′; RPL30-F 5′-GCAAAGCGAAATTGGTCATT-3′; RPL30-R 5′-CTGTTTTCACTCCTGCCACA-3′.

### *In vivo* studies

4T1 cells were treated for 14 days with water, Tat-Scr (2.5 μM) or Tat-SID (1 & 2.5 μM) and then inoculated orthotopically in the inguinal mammary gland of Balb/c mice (*n* = 5). The mice were fed ad libitum and did not receive peptide treatment. Tumor volumes were calculated as ellipsoids (Dxd^2^/2) by measuring the main diameter (D) and the smaller diameter (d) and plotted versus time (days). The experiment was stopped when tumors in the control group reached ∼500 mm^3^, then, the mice were sacrificed, tumors were isolated for weight and lungs were isolated for metastatic foci analysis. Similar experiment was also performed using MMTV-Myc cells. In another set of experiments 4T1 cells stably transfected with Scr-shRNA or PF1-shRNA were inoculated in interscapular space of Balb/c mice (*n* = 10). Tumors were surgically removed when the Scr-shRNA group reached 500 mm^3^. Tumor-free survival was calculated from Kaplan-Meier curves, and statistical significance was determined using the log-rank test for survival and the *t*-test for tumor growth. Metastatic dissemination was evaluated by dissecting the lungs from sacrificed mice and inspecting the Bouin-fixed (Sigma) lung surface for lesions using a stereoscope (Nikon SMZ800 stereoscope X3 to X5). For measuring the disseminated tumor cells in the bone marrow (BM) aspirates were collected from the bone marrow from the femurs by flushing BM with PBS plus 2X antibiotic/antimycotic (A-A) solution (Life Technologies). Red blood cells were lysed for 3 min with red cell lysis buffer (Sigma). BM cells were recovered by centrifugation (1,200 × g for 3 min) at 4°C, and re-suspended in 20 ml of culture medium with 2x A-A and 60 μM 6-thioguanine as previously described [71]. Single-cell suspensions were plated in 150 mm plated pre-coated with collagen type 1 (collagen-1 coating solution 66 μg/ml in PBS). After 24 h plates, attached cells were washed three times with PBS and fresh medium containing 6-thioguanine added. After 6 days, the number of colonies formed (each originating from a single tumor cell) were counted to evaluate the number of disseminated tumor cells (DTCs). To evaluate DTC proliferation, the number of cells per colony were counted using ImageJ software.

### Statistical analyses

Statistical analyses were performed with GraphPad Prism software (version 5.0). The experiments were conducted with at least three independent experiments unless otherwise mentioned. Where shown, p values were calculated using the unpaired Student's *t*-test, Mann-Whitney or one-way ANOVA as indicated.

## SUPPLEMENTARY MATERIAL FIGURES AND TABLES











## References

[1] Abramson VG, Mayer IA (2014). Molecular Heterogeneity of Triple Negative Breast Cancer. Curr Breast Cancer Rep.

[2] Hudis CA, Gianni L (2011). Triple-negative breast cancer: an unmet medical need. Oncologist.

[3] Arnedos M, Bihan C, Delaloge S, Andre F (2012). Triple-negative breast cancer: are we making headway at least?. Ther Adv Med Oncol.

[4] Roll JD, Rivenbark AG, Sandhu R, Parker JS, Jones WD, Carey LA, Livasy CA, Coleman WB (2013). Dysregulation of the epigenome in triple-negative breast cancers: basal-like and claudin-low breast cancers express aberrant DNA hypermethylation. Exp Mol Pathol.

[5] Tam WL, Weinberg RA (2013). The epigenetics of epithelial-mesenchymal plasticity in cancer. Nat Med.

[6] Federico M, Bagella L (2011). Histone deacetylase inhibitors in the treatment of hematological malignancies and solid tumors. J Biomed Biotechnol.

[7] Valdespino-Gomez VM, Valdespino-Castillo VE (2012). Targeted epigenetic therapy of cancer. Achievements and perspectives. Cir Cir.

[8] Ho AS, Turcan S, Chan TA (2013). Epigenetic therapy: use of agents targeting deacetylation and methylation in cancer management. Onco Targets Ther.

[9] Connolly R, Stearns V (2012). Epigenetics as a therapeutic target in breast cancer. Journal of mammary gland biology and neoplasia.

[10] Kadamb R, Mittal S, Bansal N, Batra H, Saluja D (2013). Sin3: insight into its transcription regulatory functions. Eur J Cell Biol.

[11] Bansal N, Kadamb R, Mittal S, Vig L, Sharma R, Dwarakanath BS, Saluja D (2011). Tumor suppressor protein p53 recruits human Sin3B/HDAC1 complex for down-regulation of its target promoters in response to genotoxic stress. PLoS One.

[12] Silverstein RA, Ekwall K (2005). Sin3: a flexible regulator of global gene expression and genome stability. Curr Genet.

[13] Ellison-Zelski SJ, Alarid ET (2010). Maximum growth and survival of estrogen receptor-alpha positive breast cancer cells requires the Sin3A transcriptional repressor. Mol Cancer.

[14] Ellison-Zelski SJ, Solodin NM, Alarid ET (2009). Repression of ESR1 through actions of estrogen receptor alpha and Sin3A at the proximal promoter. Mol Cell Biol.

[15] Farias EF, Petrie K, Leibovitch B, Murtagh J, Chornet MB, Schenk T, Zelent A, Waxman S (2010). Interference with Sin3 function induces epigenetic reprogramming and differentiation in breast cancer cells. Proc Natl Acad Sci U S A.

[16] Kwon YJ, Petrie K, Leibovitch BA, Zeng L, Mezei M, Howell L, Gil V, Christova R, Bansal N, Yang S, Sharma R, Ariztia EV, Frankum J, Brough R, Sbirkov Y, Ashworth A (2015). Selective inhibition of SIN3 corepressor with avermectins as a novel therapeutic strategy in triple negative breast cancer. Mol Cancer Ther.

[17] Beroukhim R, Mermel CH, Porter D, Wei G, Raychaudhuri S, Donovan J, Barretina J, Boehm JS, Dobson J, Urashima M, Mc Henry KT, Pinchback RM, Ligon AH, Cho YJ, Haery L, Greulich H (2010). The landscape of somatic copy-number alteration across human cancers. Nature.

[18] Jonsson G, Staaf J, Vallon-Christersson J, Ringner M, Holm K, Hegardt C, Gunnarsson H, Fagerholm R, Strand C, Agnarsson BA, Kilpivaara O, Luts L, Heikkila P, Aittomaki K, Blomqvist C, Loman N (2010). Genomic subtypes of breast cancer identified by array-comparative genomic hybridization display distinct molecular and clinical characteristics. Breast Cancer Res.

[19] Ayer DE, Lawrence QA, Eisenman RN (1995). Mad-Max transcriptional repression is mediated by ternary complex formation with mammalian homologs of yeast repressor Sin3. Cell.

[20] Yochum GS, Ayer DE (2001). Pf1, a novel PHD zinc finger protein that links the TLE corepressor to the mSin3A-histone deacetylase complex. Mol Cell Biol.

[21] Swanson KA, Knoepfler PS, Huang K, Kang RS, Cowley SM, Laherty CD, Eisenman RN, Radhakrishnan I (2004). HBP1 and Mad1 repressors bind the Sin3 corepressor PAH2 domain with opposite helical orientations. Nat Struct Mol Biol.

[22] Le Guezennec X, Vermeulen M, Stunnenberg HG (2006). Molecular characterization of Sin3 PAH-domain interactor specificity and identification of PAH partners. Nucleic Acids Res.

[23] Yochum GS, Ayer DE (2002). Role for the mortality factors MORF4, MRGX, and MRG15 in transcriptional repression via associations with Pf1, mSin3A, and Transducin-Like Enhancer of Split. Mol Cell Biol.

[24] Malovannaya A, Lanz RB, Jung SY, Bulynko Y, Le NT, Chan DW, Ding C, Shi Y, Yucer N, Krenciute G, Kim BJ, Li C, Chen R, Li W, Wang Y, O'Malley BW (2011). Analysis of the human endogenous coregulator complexome. Cell.

[25] Moshkin YM, Kan TW, Goodfellow H, Bezstarosti K, Maeda RK, Pilyugin M, Karch F, Bray SJ, Demmers JA, Verrijzer CP (2009). Histone chaperones ASF1 and NAP1 differentially modulate removal of active histone marks by LID-RPD3 complexes during NOTCH silencing. Mol Cell.

[26] Barrett A, Santangelo S, Tan K, Catchpole S, Roberts K, Spencer-Dene B, Hall D, Scibetta A, Burchell J, Verdin E, Freemont P, Taylor-Papadimitriou J (2007). Breast cancer associated transcriptional repressor PLU-1/JARID1B interacts directly with histone deacetylases. Int J Cancer.

[27] Catchpole S, Spencer-Dene B, Hall D, Santangelo S, Rosewell I, Guenatri M, Beatson R, Scibetta AG, Burchell JM, Taylor-Papadimitriou J (2011). PLU-1/JARID1B/KDM5B is required for embryonic survival and contributes to cell proliferation in the mammary gland and in ER+ breast cancer cells. Int J Oncol.

[28] Mitra D, Das PM, Huynh FC, Jones FE (2011). Jumonji/ARID1 B (JARID1B) protein promotes breast tumor cell cycle progression through epigenetic repression of microRNA let-7e. J Biol Chem.

[29] Vire E, Curtis C, Davalos V, Git A, Robson S, Villanueva A, Vidal A, Barbieri I, Aparicio S, Esteller M, Caldas C, Kouzarides T (2014). The breast cancer oncogene EMSY represses transcription of antimetastatic microRNA miR-31. Mol Cell.

[30] Yamamoto S, Wu Z, Russnes HG, Takagi S, Peluffo G, Vaske C, Zhao X, Moen Vollan HK, Maruyama R, Ekram MB, Sun H, Kim JH, Carver K, Zucca M, Feng J, Almendro V (2014). JARID1B is a luminal lineage-driving oncogene in breast cancer. Cancer Cell.

[31] Kumar GS, Xie T, Zhang Y, Radhakrishnan I (2011). Solution structure of the mSin3A PAH2-Pf1 SID1 complex: a Mad1/Mxd1-like interaction disrupted by MRG15 in the Rpd3S/Sin3S complex. J Mol Biol.

[32] Guo W, Keckesova Z, Donaher JL, Shibue T, Tischler V, Reinhardt F, Itzkovitz S, Noske A, Zurrer-Hardi U, Bell G, Tam WL, Mani SA, van Oudenaarden A, Weinberg RA (2012). Slug and Sox9 cooperatively determine the mammary stem cell state. Cell.

[33] Ricardo S, Vieira AF, Gerhard R, Leitao D, Pinto R, Cameselle-Teijeiro JF, Milanezi F, Schmitt F, Paredes J (2011). Breast cancer stem cell markers CD44, CD24 and ALDH1: expression distribution within intrinsic molecular subtype. J Clin Pathol.

[34] Charafe-Jauffret E, Ginestier C, Iovino F, Wicinski J, Cervera N, Finetti P, Hur MH, Diebel ME, Monville F, Dutcher J, Brown M, Viens P, Xerri L, Bertucci F, Stassi G, Dontu G (2009). Breast cancer cell lines contain functional cancer stem cells with metastatic capacity and a distinct molecular signature. Cancer Res.

[35] Azzam DJ, Zhao D, Sun J, Minn AJ, Ranganathan P, Drews-Elger K, Han X, Picon-Ruiz M, Gilbert CA, Wander SA, Capobianco AJ, El-Ashry D, Slingerland JM (2013). Triple negative breast cancer initiating cell subsets differ in functional and molecular characteristics and in gamma-secretase inhibitor drug responses. EMBO Mol Med.

[36] Ginestier C, Hur MH, Charafe-Jauffret E, Monville F, Dutcher J, Brown M, Jacquemier J, Viens P, Kleer CG, Liu S, Schott A, Hayes D, Birnbaum D, Wicha MS, Dontu G (2007). ALDH1 is a marker of normal and malignant human mammary stem cells and a predictor of poor clinical outcome. Cell Stem Cell.

[37] Sheridan C, Kishimoto H, Fuchs RK, Mehrotra S, Bhat-Nakshatri P, Turner CH, Goulet R, Badve S, Nakshatri H (2006). CD44+/CD24- breast cancer cells exhibit enhanced invasive properties: an early step necessary for metastasis. Breast Cancer Res.

[38] Honeth G, Bendahl PO, Ringner M, Saal LH, Gruvberger-Saal SK, Lovgren K, Grabau D, Ferno M, Borg A, Hegardt C (2008). The CD44+/CD24- phenotype is enriched in basal-like breast tumors. Breast Cancer Res.

[39] Meyer MJ, Fleming JM, Lin AF, Hussnain SA, Ginsburg E, Vonderhaar BK (2010). CD44posCD49fhiCD133/2hi defines xenograft-initiating cells in estrogen receptor-negative breast cancer. Cancer Res.

[40] To K, Fotovati A, Reipas KM, Law JH, Hu K, Wang J, Astanehe A, Davies AH, Lee L, Stratford AL, Raouf A, Johnson P, Berquin IM, Royer HD, Eaves CJ, Dunn SE (2010). Y-box binding protein-1 induces the expression of CD44 and CD49f leading to enhanced self-renewal, mammosphere growth, and drug resistance. Cancer Res.

[41] Strobl-Mazzulla PH, Bronner ME (2012). A PHD12-Snail2 repressive complex epigenetically mediates neural crest epithelial-to-mesenchymal transition. J Cell Biol.

[42] Shiozawa Y, Eber MR, Berry JE, Taichman RS (2015). Bone marrow as a metastatic niche for disseminated tumor cells from solid tumors. Bonekey Rep.

[43] Hartkopf AD, Stefanescu D, Wallwiener M, Hahn M, Becker S, Solomayer EF, Fehm TN, Brucker SY, Taran FA (2014). Tumor cell dissemination to the bone marrow and blood is associated with poor outcome in patients with metastatic breast cancer. Breast Cancer Res Treat.

[44] Jelinic P, Pellegrino J, David G (2011). A novel mammalian complex containing Sin3B mitigates histone acetylation and RNA polymerase II progression within transcribed loci. Mol Cell Biol.

[45] Roesch A, Fukunaga-Kalabis M, Schmidt EC, Zabierowski SE, Brafford PA, Vultur A, Basu D, Gimotty P, Vogt T, Herlyn M (2010). A temporarily distinct subpopulation of slow-cycling melanoma cells is required for continuous tumor growth. Cell.

[46] Enkhbaatar Z, Terashima M, Oktyabri D, Tange S, Ishimura A, Yano S, Suzuki T (2013). KDM5B histone demethylase controls epithelial-mesenchymal transition of cancer cells by regulating the expression of the microRNA-200 family. Cell Cycle.

[47] Kidder BL, Hu G, Zhao K (2014). KDM5B focuses H3K4 methylation near promoters and enhancers during embryonic stem cell self-renewal and differentiation. Genome Biol.

[48] Krishnakumar R, Kraus WL (2010). PARP-1 regulates chromatin structure and transcription through a KDM5B-dependent pathway. Mol Cell.

[49] Zou MR, Cao J, Liu Z, Huh SJ, Polyak K, Yan Q (2014). Histone demethylase jumonji AT-rich interactive domain 1B (JARID1B) controls mammary gland development by regulating key developmental and lineage specification genes. J Biol Chem.

[50] Baymaz HI, Karemaker ID, Vermeulen M (2015). Perspective on unraveling the versatility of ‘co-repressor’ complexes. Biochim Biophys Acta.

[51] Lee N, Erdjument-Bromage H, Tempst P, Jones RS, Zhang Y (2009). The H3K4 demethylase lid associates with and inhibits histone deacetylase Rpd3. Mol Cell Biol.

[52] Johnsen SA, Subramaniam M, Janknecht R, Spelsberg TC (2002). TGFbeta inducible early gene enhances TGFbeta/Smad-dependent transcriptional responses. Oncogene.

[53] Johnsen SA, Subramaniam M, Katagiri T, Janknecht R, Spelsberg TC (2002). Transcriptional regulation of Smad2 is required for enhancement of TGFbeta/Smad signaling by TGFbeta inducible early gene. Journal of cellular biochemistry.

[54] Dave B, Mittal V, Tan NM, Chang JC (2012). Epithelial-mesenchymal transition, cancer stem cells and treatment resistance. Breast Cancer Res.

[55] Blick T, Hugo H, Widodo E, Waltham M, Pinto C, Mani SA, Weinberg RA, Neve RM, Lenburg ME, Thompson EW (2010). Epithelial mesenchymal transition traits in human breast cancer cell lines parallel the CD44(hi/) CD24 (lo/−) stem cell phenotype in human breast cancer. J Mammary Gland Biol Neoplasia.

[56] Croker AK, Goodale D, Chu J, Postenka C, Hedley BD, Hess DA, Allan AL (2009). High aldehyde dehydrogenase and expression of cancer stem cell markers selects for breast cancer cells with enhanced malignant and metastatic ability. J Cell Mol Med.

[57] Vesuna F, Lisok A, Kimble B, Raman V (2009). Twist modulates breast cancer stem cells by transcriptional regulation of CD24 expression. Neoplasia.

[58] Muenst S, Daster S, Obermann EC, Droeser RA, Weber WP, von Holzen U, Gao F, Viehl C, Oertli D, Soysal SD (2013). Nuclear expression of snail is an independent negative prognostic factor in human breast cancer. Dis Markers.

[59] Peinado H, Ballestar E, Esteller M, Cano A (2004). Snail mediates E-cadherin repression by the recruitment of the Sin3A/histone deacetylase 1 (HDAC1)/HDAC2 complex. Mol Cell Biol.

[60] Batlle E, Sancho E, Franci C, Dominguez D, Monfar M, Baulida J, Garcia De Herreros A (2000). The transcription factor snail is a repressor of E-cadherin gene expression in epithelial tumour cells. Nat Cell Biol.

[61] Dhasarathy A, Kajita M, Wade PA (2007). The transcription factor snail mediates epithelial to mesenchymal transitions by repression of estrogen receptor-alpha. Mol Endocrinol.

[62] Bosch A, Bertran SP, Lu Y, Garcia A, Jones AM, Dawson MI, Farias EF (2012). Reversal by RARalpha agonist Am580 of c-Myc-induced imbalance in RARalpha/RARgamma expression during MMTV-Myc tumorigenesis. Breast Cancer Res.

[63] Stewart TA, Pattengale PK, Leder P (1984). Spontaneous mammary adenocarcinomas in transgenic mice that carry and express MTV/myc fusion genes. Cell.

[64] Debnath J, Muthuswamy SK, Brugge JS (2003). Morphogenesis and oncogenesis of MCF-10A mammary epithelial acini grown in three-dimensional basement membrane cultures. Methods.

[65] Gaspar-Maia A, Qadeer ZA, Hasson D, Ratnakumar K, Leu NA, Leroy G, Liu S, Costanzi C, Valle-Garcia D, Schaniel C, Lemischka I, Garcia B, Pehrson JR, Bernstein E (2013). MacroH2A histone variants act as a barrier upon reprogramming towards pluripotency. Nat Commun.

[66] Mendoza-Parra MA, Van Gool W, Mohamed Saleem MA, Ceschin DG, Gronemeyer H (2013). A quality control system for profiles obtained by ChIP sequencing. Nucleic Acids Res.

[67] Langmead B, Trapnell C, Pop M, Salzberg SL (2009). Ultrafast and memory-efficient alignment of short DNA sequences to the human genome. Genome Biol.

[68] Zhang Y, Liu T, Meyer CA, Eeckhoute J, Johnson DS, Bernstein BE, Nusbaum C, Myers RM, Brown M, Li W, Liu XS (2008). Model-based analysis of ChIP-Seq (MACS). Genome Biol.

[69] Zang C, Schones DE, Zeng C, Cui K, Zhao K, Peng W (2009). A clustering approach for identification of enriched domains from histone modification ChIP-Seq data. Bioinformatics.

[70] Liu T, Ortiz JA, Taing L, Meyer CA, Lee B, Zhang Y, Shin H, Wong SS, Ma J, Lei Y, Pape UJ, Poidinger M, Chen Y, Yeung K, Brown M, Turpaz Y (2011). Cistrome: an integrative platform for transcriptional regulation studies. Genome Biol.

[71] Aslakson CJ, Miller FR (1992). Selective events in the metastatic process defined by analysis of the sequential dissemination of subpopulations of a mouse mammary tumor. Cancer Res.

