# Supplementary material for: Targeting the SIN3A-PF1 interaction inhibits epithelial to mesenchymal transition and maintenance of a stem cell phenotype in triple negative breast cancer
Source: Oncotarget. 2015 Oct 9;6(33):34087–105. doi: 10.18632/oncotarget.6048 (PMC4741438; doi:10.18632/oncotarget.6048)
Supplement: Supplementary file 1 [file oncotarget-06-34087-s001.pdf]

Targeting the SIN3A-PF1 interaction inhibits epithelial to mesenchymal transition and maintenance of a stem cell phenotype in triple negative breast cancer

Supplementary Material

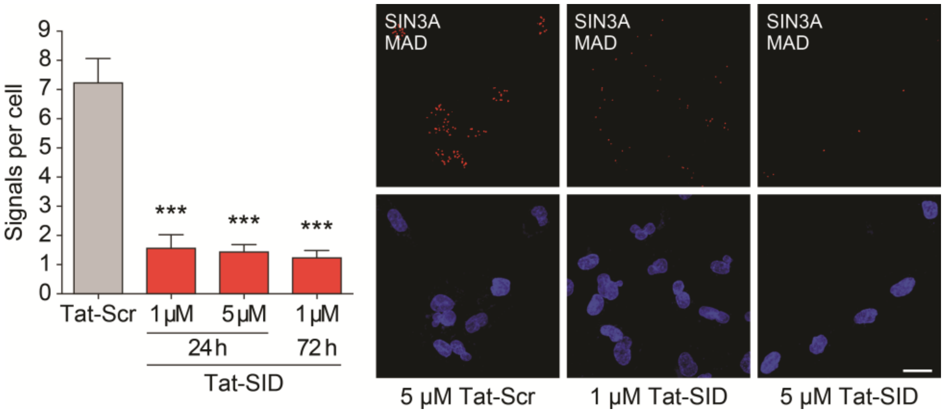

Figure S1: Tat-SID disrupts interaction between PAH2 domain of SIN3A and MAD.

Left, quantified results for proximity ligation assay (PLA) for interaction between SIN3A and MAD in 1 µM and 5 µM Tat-SID-treated MDA-MB-231 cells at 24 and 72 h compared to 5 µM Tat-Scr-treated cells. Tat-Scr versus 1 µM Tat-SID 24 h, ***, $p = 0.0005$; Tat-Scr versus 5 µM Tat-SID 24 h, ***, $p = 0.0003$; Tat-Scr versus 1 µM Tat-SID 72 h, ***, $p = 0.0003$; p, unpaired t-test. Error bars represent mean ± SD ($n = 3$). Right, PLA analyzing SIN3A:MAD interactions following Tat-SID or Tat-Scr treatment at the indicated concentrations (24 h). Red dots represent the presence of SIN3A:MAD interactions. Dots may only be generated when antibody-conjugated PLA probes are in close proximity (< 40 nm), permitting ligation, amplification and detection with complementary fluorescent probes. Antibodies used are indicated by white text. Scale bar indicates 20 µM.

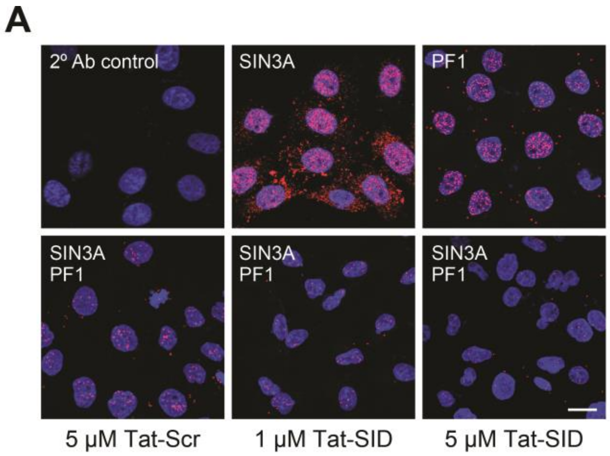

B

Peptide	IC_{50}
MAD-SID	1.26 μM
MAD-Scr	Not detectable
PF1-SID	15.59 μM
PF1-Scr	Not detectable

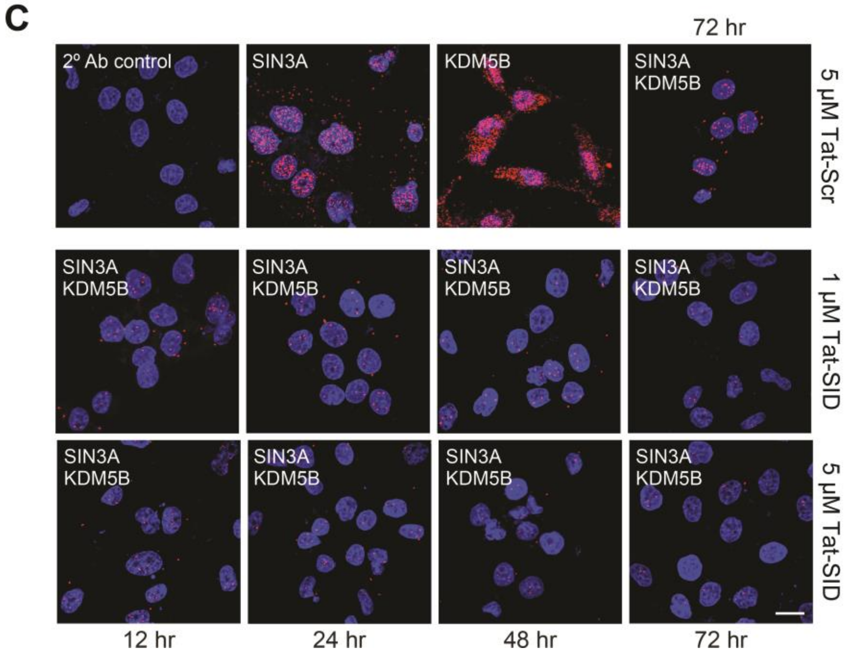

Figure S2: Tat-SID disrupts interaction between PAH2 domain of SIN3A and PF1 and KDM5B

(A) PLA analysis of SIN3A/PF1 interactions following Tat-SID or Tat-Scr treatment at the indicated concentrations treatment (24 h). Red dots represent expression of SIN3A, PF1 or the presence SIN3A:PF1 interactions as indicated.

(B) Binding affinity of MAD-SID and PF1-SID for the PAH2 domain of SIN3A determined by fluorescence polarization competition assay using a FITC-conjugated MAD peptide as a probe.

(C) PLA analysis of SIN3A:KDM5B interactions following Tat-SID or Tat-Scr treatment at the indicated concentrations and times. Red dots represent expression of SIN3A, KDM5B or the presence SIN3A:KDM5B interactions as indicated. Antibodies used are indicated by white text (2º Ab, secondary antibody control). Scale bar indicates 20 µM.

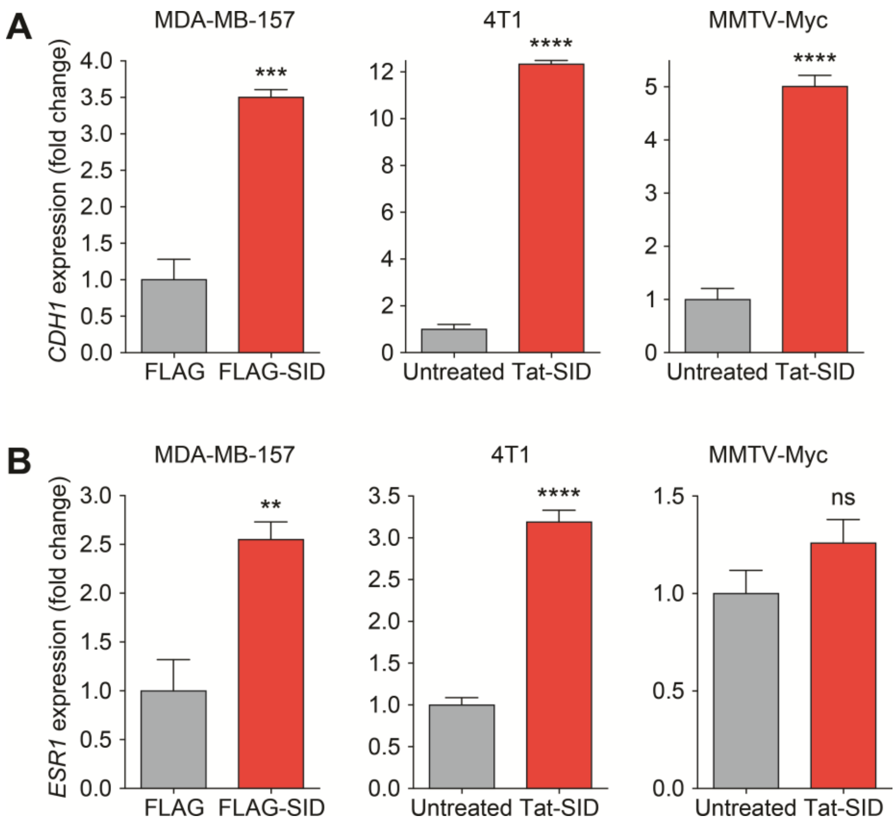

Figure S3: Tat-SID treatment re-expresses *CDH1* and *ESR1* in TNBC lines.

(A) qRT-PCR for *CDH1* in MDA-MB-157 cells stably transfected with FLAG-SID, or 4T1 or MMTV-Myc cells treated with 2.5 μM Tat-SID for 72 h as indicated. MDA-MB-157, ***, $p = 0.0001$; 4T1, ****, $p < 0.0001$; MMTV-Myc, ****, $p < 0.0001$.

(B) qRT-PCR for *ESR1* in MDA-MB-157 cells stably transfected with FLAG-SID, or 4T1 or MMTV-Myc cells treated with 2.5 μM Tat-SID for 72 h as indicated. MDA-MB-157, **, $p = 0.0019$; 4T1, ****, $p < 0.0001$; MMTV-Myc, ns, $p < 0.0568$.

Error bars represent mean ± SD ($n = 3$). p, unpaired t-test.

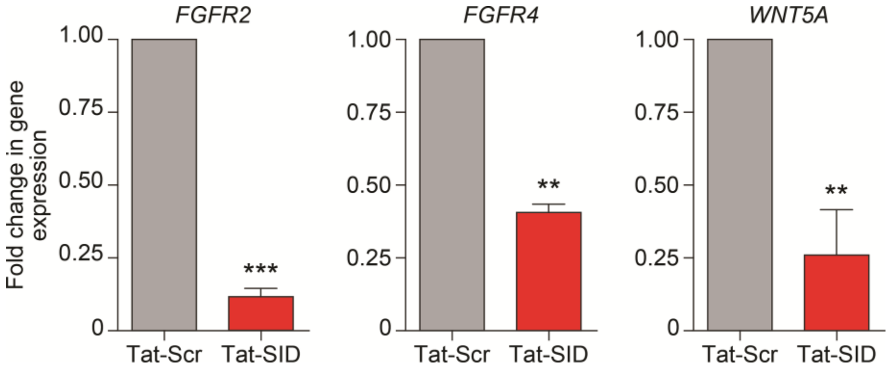

Figure S4: Tat-SID down-regulates expression of genes functioning in epithelial-mesenchymal transition.

(A) qRT-PCR for *FGFR2*, *FGFR4* and *WNT5A* in MDA-MB-231 cells treated with 1 μM Tat-Scr or Tat-SID for 72 h (*FGFR2* and *FGFR4*) or 24 h (*WNT5A*). *FGFR2*, Tat-Scr versus Tat-SID, ***, p = 0.0005; *FGFR4*, Tat-Scr versus Tat-SID, **, p = 0.00011; *WNT5A*, Tat-Scr versus Tat-SID, **, p = 0.00012; p, one sample t-test. Error bars represent mean ± SD (n = 3).

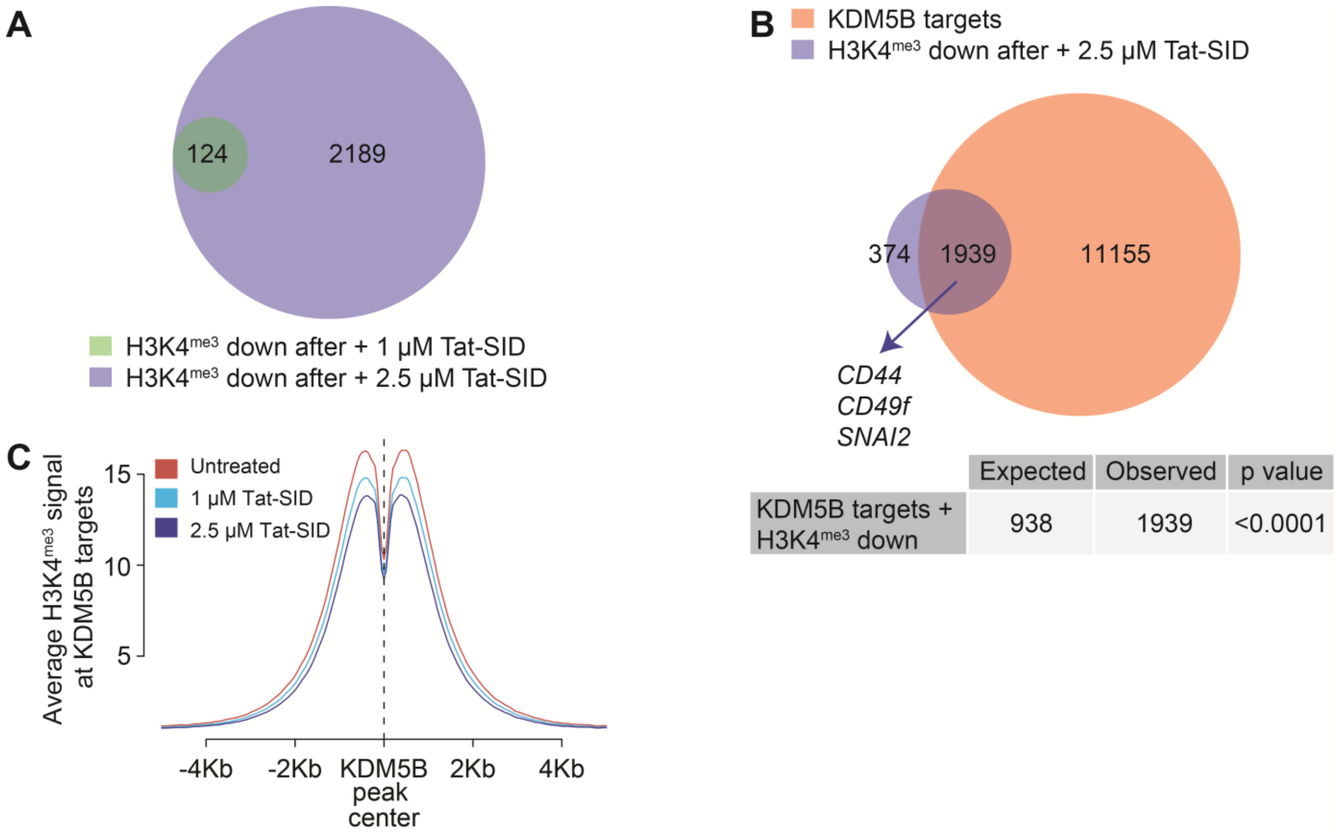

Figure S5: Tat-SID decreases global promoter H3K4 trimethylation.

(A) Venn diagram representing genes with significantly reduced H3K4me^3 level at promoter (FDR < 1x10-15) after SID treatment in MDA-MB-231 cells.

(B) Venn diagram representing overlap between genes with H3K4me^3 reduction and KDM5B bound genes [1] in MDA-MB-231 cells.

(C) Average H3K4me^3 ChIP signal around KDM5B peak center (-5Kb to +5Kb) [1] in untreated and SID treated MDA-MB-231 cells.

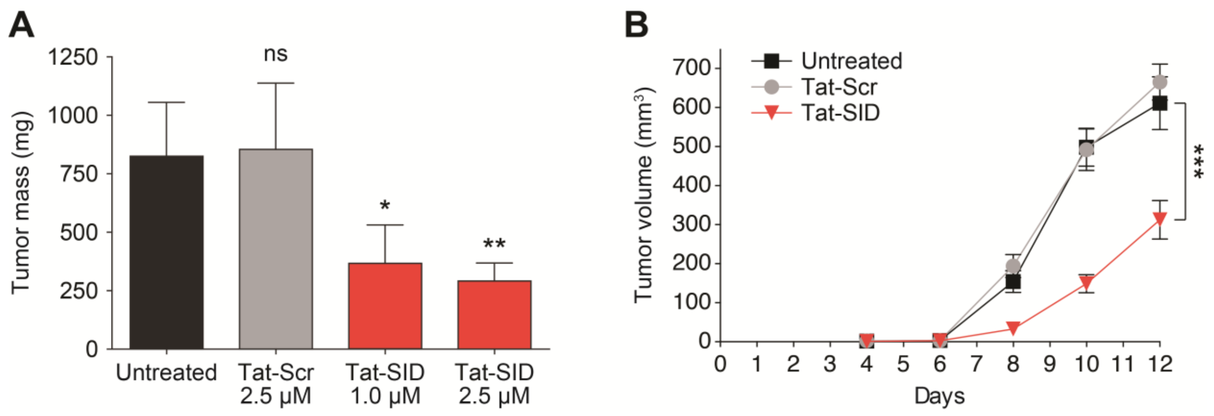

Figure S6: Ex vivo Tat-SID treatment inhibits tumor progression in mouse models of TNBC.

(A) Tumor mass in Balb/c mice (n = 5) after 20 days from injection of 4T1 cells treated with water, Tat-Scr (2.5 µM) or Tat-SID (1 µM and 2.5 µM). Tat-Scr versus 1 µM Tat-SID, *, p = 0.0101; Tat-Scr versus 2.5 µM Tat-SID, **, p = 0.0026; p, unpaired t-test.

(B) Tumor progression in FVB mice (n = 5) injected with MMTV-Myc cells treated with Tat-SID or Tat-Scr for 7 days. Tat-Scr versus Tat-SID,***, p = 0.0001; p, one way ANOVA.

Error bars represent mean ± SD.

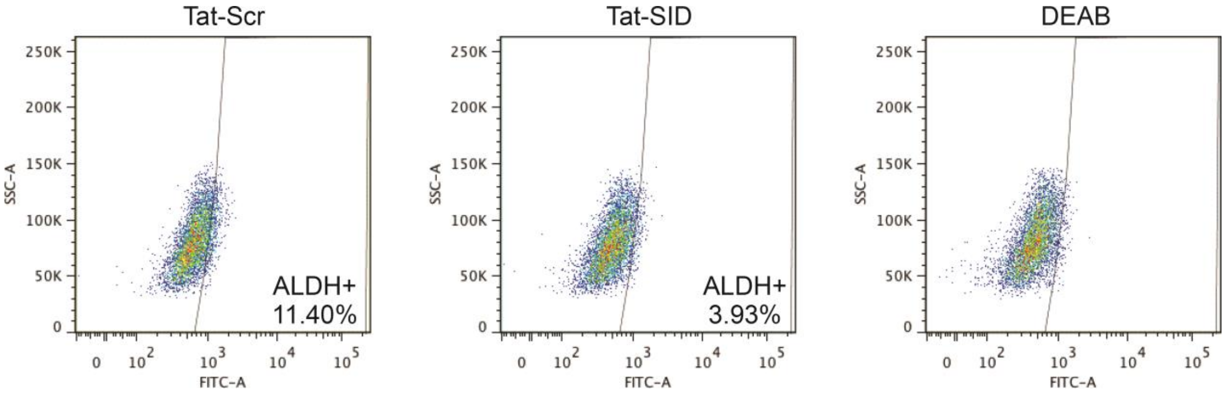

Figure S7: FACS analysis of ALDH1 activity in 4T1 cells.

Cells were treated with 1 μM Tat-Scr or Tat-SID for 14 days. Results are quantified as percentage of ALDH+ cells.

Supplementary Table 2: IPA analysis for significantly enriched cellular functions in MDA-MB-231 cells treated with Tat-SID	
Cellular Function or Pathway	p value
Regulation of Epithelial-Mesenchymal Transition	2.48E-04
Cellular Growth and Proliferation	1.72E-12
Cell Migration	1.12E-10
Cell Death and Survival	5.76E-11

Supplementary Table 5: Upstream Regulator Analysis from IPA	
Transcription Factor	Z Score
TGFB1	-4.4
PDGF BB	-3.2
CTNNB1	-3.3
SMAD3	-2.6
SMAD4	-2.2
NFkB (complex)	-2.9
E2F1	-2.1
ERK	-2.8
RARG	-2.0

Supplementary Table 7: ChIP-seq Summary				
	H3K4^{me3} Down (FDR<q1e-15)		H3K4^{me3} Up (FDR<q1e-15)	
	Regions	Genes	Regions	Genes
SID1/UT	141	124	0	0
SID2.5/UT	2063	2313	3	0

Supplementary Table 8: Primers for qRT-PCR		
Gene	Fwd Sequence (5'-3')	Rev Sequence (5'-3')
CDH1	CCGCTGGCGTCTGTAGGA	AGGGCTCTTTGACCACCGCTCT
ESR1	GATCCACCTGATGGCCAAG	ACAGATGCTCCATGCCTTTG
NANOG	TTTGTGGGCCTGAAGAAAACT	AGGGCTGTCCTGAATAAGCAG
OCT4	CTTGAATCCCGAATGGAAAG	GTGTATATCCCAGGGTGATC
SOX2	TACAGCATGTCCTACTCGCAG	GAGGAAGAGGTAACCACAGGG
RPL30	GACAAGGCAAAGCGAAATTG	GTATTTTCCGCATGCTGTGC

Supplementary Table 9: Summary of ChIP-Seq Quality Control				
	UT - Input	UT - H3K4^{me3}	SID (1µM) - H3K4^{me3}	SID (2.5µM) - H3K4^{me3}
Total seq reads (x10^{6})	127	32	40	30
Duplication (%)	24.9	55.9	55	48.6
Reads aligned (%)	86.7	84.8	84.9	89.8
Reads unaligned by -m<20 (%)	2	0.5	0.6	0.8
Unique reads (%)	88.7	57.1	57.9	64.3
Total unique aligned reads (x10^{6})	97.7	15.5	19.5	17.6
QC stamp	AAA	AAB	AAB	AAB

UT, untreated; SID, Tat-SID treatment

Supplementary References

1. Yamamoto S, Wu Z, Russnes HG, Takagi S, Peluffo G, Vaske C, Zhao X, Moen Vollan HK, Maruyama R, Ekram MB, Sun H, Kim JH, Carver K, Zucca M, Feng J, Almendro V, et al. JARID1B is a luminal lineage-driving oncogene in breast cancer. Cancer Cell. 2014; 25(6):762-777.
